# Targeting E3 ubiquitin ligases: Mechanistic breakthroughs and novel clinical translation pathways for tumor radioimmunotherapy

**DOI:** 10.1186/s12943-026-02739-x

**Published:** 2026-07-22

**Authors:** Qian Yang, Xinruo Xing, Yichun Wang, Xiangyi Wang, Yutong Han, Jiaxin Ren, Zhenyong Zhang, Jun Tang, Jianzhu Zhao

**Affiliations:** 1 Department of Oncology, Shengjing Hospital of China Medical University, Shenyang, Liaoning P. R. China; 2 Department of Thoracic Surgery, Shengjing Hospital of China Medical University, Shenyang, Liaoning P. R. China

**Keywords:** E3 ubiquitin ligases, Radioimmunotherapy, Ubiquitination, Radiotherapy, Immunotherapy, Regulatory mechanism, Clinical application

## Abstract

The combination of radiation therapy and immunotherapy has become a cornerstone of modern clinical cancer treatment. However, the inherent radiation resistance of tumors and complex immune evasion mechanisms remain major bottlenecks limiting their long-term effects and sustained efficacy. E3 ubiquitin ligases critically influence tumor sensitivity to radioimmunotherapy by controlling protein stability across DNA repair, immune signaling, and stress-response pathways. This review systematically dissects the multidimensional molecular network through which E3 ubiquitin ligases regulate radiosensitivity and immunoresponsiveness. At the intracellular level, we provide an in-depth analysis of how E3 ubiquitin ligases determine the fate of radiation-induced damage repair by precisely regulating the kinetics of the DNA damage response (DDR), cell cycle checkpoints, and apoptosis thresholds. At the extracellular level, this study focuses on the key roles of E3 ubiquitin ligases in reshaping the immune microenvironment, including the maintenance of spatiotemporal stability of immune checkpoints, the fidelity of antigen processing and presentation, and the epigenetic regulation of microenvironmental dynamic plasticity. Recent studies indicate that E3 ubiquitin ligases link radiation-induced DDR signaling to innate and adaptive immune activation, particularly through the induction of immunogenic cell death (ICD) and the calibration of innate immune sensing pathways like cGAS-STING. Finally, we provide a comprehensive synthesis of cutting-edge translational strategies targeting E3 ubiquitin ligases—ranging from canonical inhibitors to transformative proteolysis-targeting chimeras (PROTACs) and molecular glue degraders (MGDs)—offering novel paradigms for overcoming therapeutic resistance and refining personalized radioimmunotherapy.

## Introduction

Radiotherapy damages the DNA of tumor cells with high-energy rays to precisely eliminate lesions [1]. Meanwhile, it can reshape the TME via the in situ vaccine effect, reduce the proportion of immunosuppressive cells such as Tregs and MDSCs, and downregulate the expression of immune checkpoint molecules including PD-L1, enhancing the efficacy of immunotherapy [2]. The core of immunotherapy is to activate the body’s immune system and break immune tolerance. It not only strengthens the antitumor immune response but also amplifies the effect of radiotherapy, eliminates residual micrometastases and distant metastases after radiotherapy, and achieves a synergistic outcome of “local treatment triggering systemic immunity“ [2–4]. Currently, the combined application of radioimmunotherapy has attracted considerable attention.

However, the efficacy of radiotherapy and immunotherapy is influenced by multiple factors [5]. The outcome of radiotherapy depends on the patient’s physiological state (such as age, underlying disease, and immune function) as well as the intrinsic characteristics and microenvironment of the tumor, including cell cycle changes [6], apoptosis [7], and DDR [8]. Immunotherapy relies on the state of the immune system, the immunogenicity of the tumor, and the expression levels of checkpoint proteins such as PD-1 and CTLA-4 [9].

Notably, the antitumor effect of radiotherapy and the regulation of immune response in immunotherapy both depend on an elaborate dynamic protein regulatory network. The ubiquitin–proteasome system (UPS) governs protein turnover and has emerged as an important regulator of therapeutic response in cancer [10]. By imposing specific ubiquitination modifications on substrate proteins, the UPS is critically involved in the pathological processes of uncontrolled tumor cell proliferation, blocked apoptosis, and host immune evasion; its regulatory efficacy determines the lethality of radiation therapy against DNA damage and the upper limit of immune therapy’s ability to activate antitumor immunity [11]. E3 ubiquitin ligases are classified into three distinct subtypes on the basis of their structural characteristics. One is homologous to the C-terminus of the E6-associated protein (HECT), the other contains the interesting new gene (RING) domain, and the third has the RING-between-RING (RBR) domain [12]. Studies have confirmed that E3 ubiquitin ligases play an irreplaceable regulatory role in tumor radiosensitization and immune response activation [13]. Elucidating their underlying mechanisms can provide theoretical support for the synergistic effects of radioimmunotherapy combinations and the development of novel therapeutic strategies, which represents a key breakthrough for improving the efficacy of comprehensive tumor therapy. In this review, we focus on the ubiquitination regulatory network centered on E3 ubiquitin ligases, explore in depth their regulatory mechanisms underlying radiotherapy, immunotherapy and their combination therapy, further summarize the current progress of their clinical translation, and clarify their development value as potential therapeutic targets.

## E3 ubiquitin ligases: Independent determinants of radiosensitivity and antitumor immunity

**Fig. 1:**
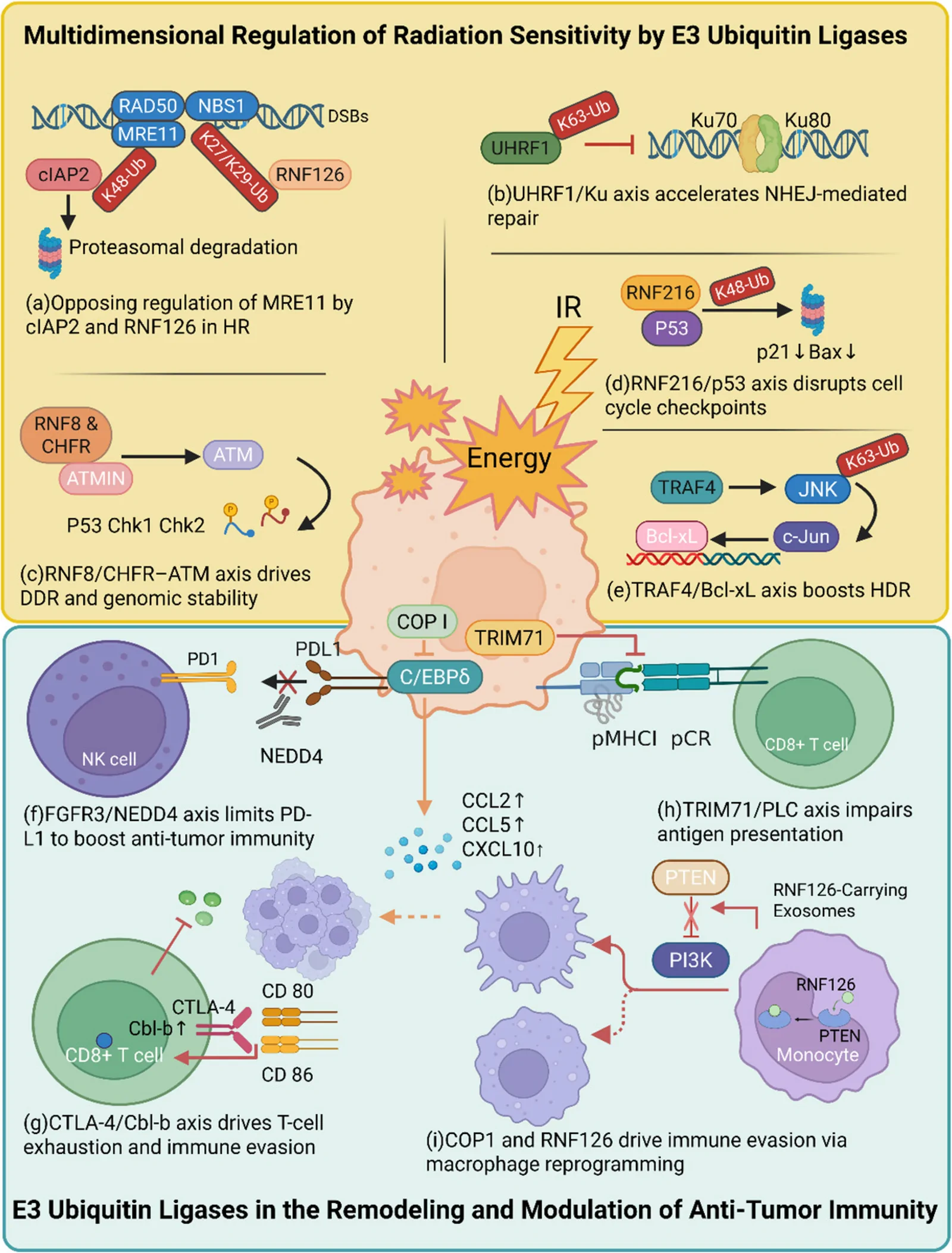
Multidimensional Regulation of Radiosensitivity and Antitumor Immunity by E3 Ubiquitin Ligases. E3 ubiquitin ligases coordinate DNA damage signaling with remodeling of the immune microenvironment. (**a**) In homologous recombination (HR), cIAP2 (cellular inhibitor of apoptosis protein 2) mediates the K48-linked proteasomal degradation of MRE11, while RNF126 (Ring Finger Protein 126) promotes non-degradative K27/K29-linked ubiquitination to enhance MRE11 activity. (**b**) UHRF1 (ubiquitin-like with PHD and ring finger domains 1) stabilizes the Ku70/Ku80 complex via K63-linked ubiquitination to accelerate non-homologous end joining (NHEJ). (**c**) RNF8 (Ring finger protein 8) and CHFR (checkpoint protein with FHA and RING domains) activate ATM (Ataxia-telangiectasia mutated kinase) to drive the DNA damage response (DDR) via p53, Chk1, and Chk2 (checkpoint kinase 1 and 2). (**d**) RNF216 (Ring finger protein 216) mediates p53 degradation, downregulating p21 and Bax. (**e**) TRAF4 (TNF Receptor Associated Factor 4) activates the JNK (c-Jun N-terminal kinase)/c-Jun pathway to upregulate Bcl-xL, driving homology-directed repair (HDR). (**f**) NEDD4 (Neural precursor cell expressed developmentally downregulated 4) catalyzes the degradation of PD-L1 (programmed death-ligand 1) in FGFR3 (fibroblast growth factor receptor 3)-activated cells. (**g**) Cbl-b (Casitas B-lineage lymphoma-b) mediates the degradation of TCR (T cell receptor) components to promote immune evasion. (**h**) TRIM71 (Tripartite motif-containing protein 71) degrades the PLC (peptide loading complex), inhibiting pMHCI (peptide-MHC class I) surface expression. (**i**) COP1 (Component of photomorphogenic complex 1) degrades C/EBPδ (CCAAT/enhancer-binding protein delta), promoting M2 macrophage infiltration via chemokines like CCL2/5 and CXCL10

Before delving into the synergistic mechanisms between radiation therapy and immunotherapy, it is crucial to elucidate the intrinsic mechanisms by which E3 ubiquitin ligases independently regulate these two major therapeutic modalities. E3 ubiquitin ligases not only define the radiosensitivity of tumor cells by finely regulating DDR and cell fate-determining proteins, but also act as central regulators of membrane and cytoplasmic protein homeostasis, independently mediating tumor immune recognition and escape processes (Fig. 1; Table 1).

**Table 1:** Differential roles of E3 ubiquitin ligases in the independent regulation of tumor radiosensitivity and antitumor immunity

Regulatory Dimension	Name	Tumor type	Major mechanism	Outcome	References
Radiosensitivity	cIAP2	Bladder cancer	Mediates proteasomal degradation of MRE11, impairing homologous recombination (HR) repair.	Enhances radiosensitivity	[15, 16]
	RNF126	TNBC	Mediates non-degradative K27/K29-linked ubiquitination, enhancing MRE11 exonuclease activity.	Increases radioresistance	[17, 18]
	UHRF1	ESCC, BC	Mediates K63-linked ubiquitination to stabilize Ku complex and accelerate non-homologous end joining (NHEJ).	Increases radioresistance	[20]
	RNF8	--	Cooperates with CHFR to activate ATM, induces cell cycle arrest	Enhances radioresistance	[21, 22]
	RNF216	GBM	Promotes p53 degradation, downregulating p21 and Bax, thereby impairing cell cycle arrest and apoptosis.	Decreases radiosensitivity	[24, 25]
	TRAF4	CRC	Activates JNK/c-Jun axis to drive the transcription of the anti-apoptotic protein Bcl-xL.	Increases radioresistance	[27]
Antitumor Immunity	NEDD4	Bladder cancer	Catalyzes K48-linked ubiquitination and degradation of PD-L1 to maintain CD8⁺ T cell infiltration.	Enhances immune surveillance	[30, 31]
	Cbl-b	TNBC	Mediates degradation of TCR signaling components, blocking effector T cell activation and cytokine secretion.	Promotes immune escape	[32]
	TRIM71	TNBC	Degrades the peptide loading complex, inhibiting the surface expression of MHC-I molecules.	Impairs antigen presentation	[33]
	COP1	TNBC	Promotes C/EBPδ degradation, increasing M2 macrophage infiltration and suppressing CD8⁺ T cell function.	Shapes immunosuppressive TME	[35]
	RNF126	NPC	Promotes M2 macrophage polarization via degrading PTEN	Shapes immunosuppressive TME	[36]

### Multidimensional control of radiosensitivity: From DDR to cell fate orchestration

The efficacy of radiotherapy fundamentally depends on the dynamic balance between the repair rate of ionizing radiation (IR)-induced damage in tumor cells and the expression of cell fate-determining factors. As central regulators of protein homeostasis, E3 ubiquitin ligases exert a critical influence on radiotherapy outcomes by precisely controlling DDR signaling pathways and cell cycle checkpoints.

#### Precision modulation of the DDR landscape

DNA double-strand breaks (DSBs) represent the key target for IR-induced cell death, and their repair mainly relies on two core pathways: homologous recombination (HR) and non-homologous end joining (NHEJ) [14]. By targeting and regulating key proteins in the DDR pathways, E3 ubiquitin ligases can affect the efficiency of DSBs repair and consequently alter the radiosensitivity of tumor cells, which is currently the most extensively studied regulatory mechanism.

HR repair is a high-fidelity DNA repair pathway that relies on the synergistic action of core proteins, including the MRE11/RAD50/NBS1 (MRN) complex and RAD51 [15]. As a RING family E3 ubiquitin ligase, cellular inhibitor of apoptosis 2 (cIAP2) promotes the ubiquitin-dependent degradation of MRE11 via the proteasomal pathway in bladder cancer cells, reduces its protein level, and impairs the HR repair capacity of cells for DSBs, ultimately providing a potential target for radiosensitization in bladder cancer [16]. In triple-negative breast cancer (TNBC), Ring finger protein 126 (RNF126), via its E3 ligase activity, modifies residues K339 and K480 of MRE11 with K27/K29-linked polyubiquitin chains, which enhances the exonuclease activity of MRE11 without inducing its degradation [17]. As the activation of MRE11 is the initiating step of HR-mediated DDR [18], the interaction of RNF126 with the MRN complex can promote sustained DDR, increasing the radioresistance of TNBC cells.

NHEJ is a rapidly initiated DDR pathway, which relies on the involvement of molecules such as the Ku70/Ku80 heterodimer and the XRCC4/LigIV/XLF complex [19]. In esophageal squamous cell carcinoma (ESCC) and breast cancer (BC), ubiquitin-like with PHD and ring finger domains 1 (UHRF1) mediates non-degradative ubiquitination modification (e.g., K63-linked polyubiquitin chains) of Ku70/Ku80 via the E3 ubiquitin ligase activity of its RING. This modification enhances the binding capacity of Ku70/Ku80 to the broken ends of DSBs, accelerates the initial recognition step of the NHEJ pathway, and simultaneously inhibits the ubiquitin-dependent degradation of Ku70 to maintain its protein stability, which underpins the NHEJ repair process. Consequently, it strengthens the DDR and reduces radiosensitivity [20].

ATM kinase is a core initiating molecule in the DDR signaling pathway. It can trigger cell cycle arrest and promote the DNA repair process by phosphorylating downstream effector molecules such as Chk1/Chk2 and p53. E3 ubiquitin ligases can indirectly regulate the function of the DDR pathway through modulating the activity of ATM and its upstream and downstream molecules [21]. After the occurrence of DSBs, Ring finger protein 8 (RNF8) can cooperate with the checkpoint protein CHFR to activate ATM kinase, thus inducing the phosphorylation of target proteins including p53, Chk1/Chk2, NBS1 and BRCA1. This induces cell cycle arrest to allow sufficient time for the repair of radiation-induced DNA damage, ultimately enhancing cellular radioresistance and maintaining genomic stability [21, 22].

#### Dynamic reshaping of cell cycle progression and proliferative signaling

IR can induce significant G1, S, or G2/M phase arrests in tumor cells. This stress response provides a critical kinetic window for the repair of damaged DNA. During this process, E3 ubiquitin ligases dynamically reshape the cell cycle distribution by precisely targeting cell cycle-regulating proteins, thereby determining the post-irradiation survival fate and radiosensitivity of tumor cells [23].

Studies have demonstrated that high expression of Ring finger protein 216 (RNF216) in tumor tissues of glioblastoma multiforme (GBM) patients is significantly correlated with poor prognosis. In particular, in GBM with wild-type p53, the expression level of RNF216 can serve as a predictive indicator for radiotherapy efficacy. Further mechanistic investigations have revealed that RNF216 interacts with p53, promoting its ubiquitination and subsequent degradation. This results in reduced expression of p53 and its downstream effectors (including p21, Bax, PTEN and p48), which impairs p53-mediated cell cycle regulation, DNA repair and apoptotic pathways, ultimately reducing the radiosensitivity of GBM [24]. Preclinical studies have confirmed that the combination of RNF216 inhibitors and radiotherapy exerts a synergistic antitumor effect without obvious toxicity. Moreover, the combined application of RNF216 inhibitors with p53 activators (e.g., nutlin-3) can enhance p53 function both upstream and downstream, achieving a supra-additive radiosensitizing effect [25].

In colorectal cancer (CRC), high expression of the anti-apoptotic protein Bcl-xL can enhance the homology-directed repair (HDR) of DSBs [26]. Studies have shown that under IR conditions, TNF Receptor Associated Factor 4 (TRAF4) promotes the K63-linked ubiquitination of JNK1/2, enhances its kinase activity, activates the JNK/c-Jun signaling pathway, and upregulates c-Jun expression, driving the transcription of Bcl-xL. Therefore, TRAF4 enhances HDR capacity by upregulating Bcl-xL, which in turn reduces the radiosensitivity of CRC cells [27].

### Independent remodeling of the tumor immune landscape by E3 ubiquitin ligases

Immunotherapy primarily functions by activating host antitumor immunity, thus enabling the precise recognition and effective elimination of tumor cells [3]. Research indicates that E3 ubiquitin ligases can influence the efficacy of tumor immunotherapy through multiple pathways.

#### Proteostatic regulation of immune checkpoint signaling

Tumor cells express immune checkpoint molecules that bind to PD-1 and CTLA-4 receptors on the surface of T cells or NK cells, hence suppressing the activation of T cells or NK cells. Immune checkpoint inhibitors (ICIs) can block these signaling pathways, restoring the ability of T cells or NK cells to attack tumor cells [28].

PD-1 is a core inhibitory receptor on the surface of immune cells that mediates tumor immune escape [29]. E3 ligase Neural precursor cell expressed developmentally downregulated 4 (NEDD4) can catalyze K48 polyubiquitination and proteasomal degradation of PD-L1 in bladder cancer with fibroblast growth factor receptor 3 (FGFR3) activation, maintaining low PD-L1 expression and preserving CD8⁺ T cell infiltration and antitumor function. Inhibition of FGFR3 leads to decreased NEDD4 activity, subsequent PD-L1 accumulation and CD8⁺ T cell suppression, which impairs the efficacy of immunotherapy. This mechanism provides a molecular basis for the combined targeted therapy of FGFR3 and immunotherapy [30, 31].

The E3 ubiquitin ligase Casitas B-lineage lymphoma-b (Cbl-b) is a key downstream molecule of the CTLA-4 pathway. When CTLA-4 competitively binds to its ligands CD80/CD86, it induces an upregulation of Cbl-b expression and activates its enzymatic activity. In this activated state, Cbl-b specifically mediates the ubiquitination and degradation of core components of the T cell receptor (TCR) signaling complex, such as PI3K, PLCγ, and the TCRζ chain. This protein metabolic process directly disrupts the signaling cascade, leading to the inactivation of key transcription factors such as NF-κB and NFAT, blocking the clonal expansion and cytokine release of effector T cells at the source. Furthermore, the CTLA-4/Cbl-b axis does not operate in isolation; it can synergize with the PD-1/PD-L1 signaling axis, inducing the accumulation of exhaustion markers such as PD-1 and TIM-3, deeply consolidating the tumor’s immune evasion phenotype [32].

#### Modulating the fidelity of antigen processing and presentation

In TNBC, the E3 ubiquitin ligase Tripartite motif-containing protein 71 (TRIM71) degrades core components of the peptide loading complex (PLC) via the K48-linked ubiquitination pathway, blocking antigen peptide transport to the endoplasmic reticulum and the assembly of MHC class I molecules. This reduces MHC class I expression on tumor cell surfaces, diminishing the ability of CD8⁺ T cells to recognize tumors. Moreover, TRIM71 degrades the tumor suppressor proteins p53 and Rb via ubiquitination, further suppressing the transcription of antigen presentation-related genes and regulating tumor cell growth [33]. This dual mechanism of immune evasion plays a pivotal role in TNBC immune escape, and targeted intervention offers a potential new strategy for reversing resistance to immunotherapy.

#### Orchestrating the TME and immune cell plasticity

E3 ubiquitin ligases participate in the regulation of tumor progression and immune responses by modulating the functions and signaling pathways of TME, and their roles exhibit tumor specificity.

Component of photomorphogenic complex 1 (COP1) is a crucial E3 ubiquitin ligase that plays key roles in cell growth, immune regulation, and tumorigenesis [34]. In TNBC, COP1 mediates the ubiquitination and degradation of the transcription factor C/EBPδ via the scaffold protein Trib2. COP1 deficiency enhances C/EBPδ stability, inhibits the transcription of macrophage chemokines, reduces M2-type macrophage infiltration, abrogates the functional suppression of CD8⁺ T cells, reshapes the antitumor immune microenvironment, and simultaneously enhances tumor sensitivity to immune checkpoint blockade (ICB) therapies such as PD-1 inhibitors, exerting a synergistic effect on tumor growth inhibition [35]. Therefore, targeting COP1 provides a novel strategy for improving the efficacy of immunotherapy in TNBC.

In NPC, exosomes derived from tumor cells can deliver Ring finger protein 126 (RNF126) to macrophages. RNF126 induces the ubiquitination and degradation of the tumor suppressor protein PTEN, relieving its inhibitory effect on the PI3K/AKT pathway, which in turn impairs macrophage autophagy and promotes their polarization toward the M2 phenotype. M2-type macrophages shape an immunosuppressive microenvironment by regulating the secretion of anti-inflammatory/pro-inflammatory cytokines, driving tumor proliferation, epithelial-mesenchymal transition (EMT) and metastasis, as well as reducing the efficacy of immunotherapy [36].

## E3 ubiquitin ligases: The molecular hub integrating radiation therapy and the tumor immune response

**Fig. 2:**
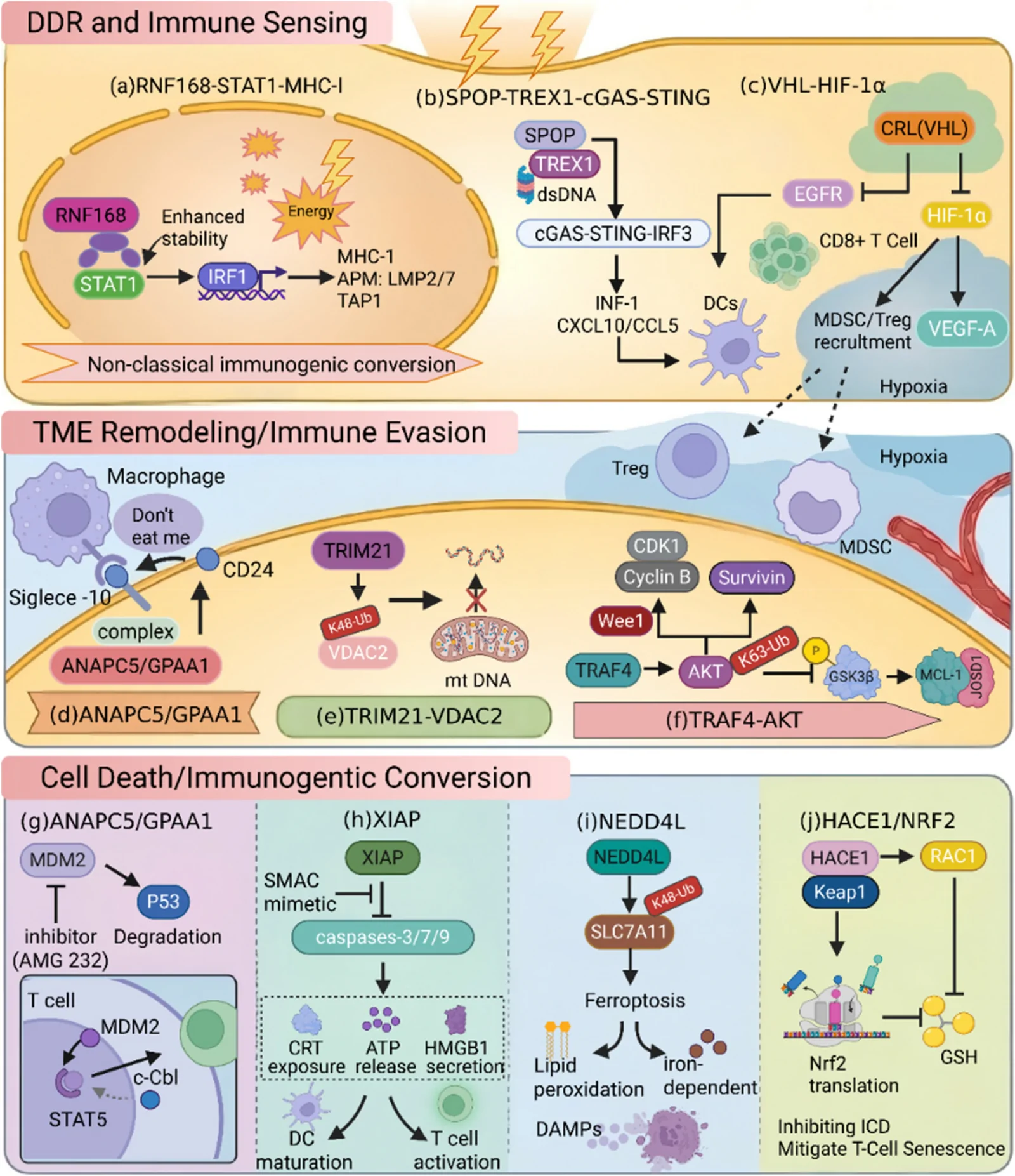
E3 Ubiquitin Ligases as Integrative Hubs for Radiation-Induced Immune Response and TME Remodeling. E3 ubiquitin ligases integrate physical radiation damage with immunobiological responses by precisely regulating protein homeostasis. (**a**) RNF168 (Ring finger protein 168) stabilizes STAT1 (Signal transducer and activator of transcription 1) to drive IRF1 (Interferon regulatory factor 1)-mediated expression of MHC-I (Major histocompatibility complex class I) and APM (Antigen processing machinery: LMP2/7, TAP1). (**b**) SPOP (Speckle-type POZ protein) degrades TREX1 to lower the cGAS-STING (Cyclic GMP-AMP synthase-Stimulator of interferon genes) activation threshold, inducing IFN-I (Type I interferon), CXCL10, and CCL5. (**c**) CRL (Cullin-RING E3 ligase) subunit VHL (Von Hippel-Lindau) degrades EGFR and HIF-1α (Hypoxia-inducible factor-1α) to suppress VEGF-A (Vascular endothelial growth factor A) and the recruitment of MDSCs (Myeloid-derived suppressor cells) and Tregs (Regulatory T cells). (**d**) ANAPC5 (Anaphase-promoting complex subunit 5) facilitates CD24 transport, sending “don’t eat me” signals to macrophages via Siglec-10. (**e**) TRIM21 (Tripartite Motif-containing Protein 21) degrades VDAC2 to sequester mtDNA (Mitochondrial DNA), dampening innate sensing. (**f**) TRAF4 (TNF Receptor Associated Factor 4) establishes radioresistance via AKT-mediated stabilization of Survivin and MCL-1. (**g**) MDM2 (Mouse double-minute 2) degrades p53 in tumors but stabilizes STAT5 in T cells to sustain antitumor immunity. (**h**) Inhibiting XIAP (X-linked inhibitor of apoptosis) via SMAC mimetics shifts death to ICD (Immunogenic cell death), releasing DAMPs (Damage-associated molecular patterns: CRT, ATP, HMGB1) for DC (Dendritic cell) maturation. (**i**) NEDD4L (Neural precursor cell-derived downregulated protein 4-like) degrades SLC7A11 (Solute Carrier Family 7 Member 11) to amplify radiation-induced ferroptosis. (**j**) HACE1 (HECT domain and Ankyrin repeat-containing E3 ubiquitin-protein ligase 1) regulates redox via Keap1-Nrf2 and RAC1 to modulate ICD and mitigate T-cell senescence

Although E3 ubiquitin ligases independently regulate radiosensitivity and antitumor immunity, accumulating studies indicate that many of these ligases also connect radiation-induced DNA damage signaling with immune activation pathways. Following irradiation, E3 ubiquitin ligases modulate multiple processes involved in antitumor immunity, including cytosolic DNA sensing, interferon signaling, antigen presentation, and immune checkpoint stability. Through these mechanisms, ubiquitin-dependent signaling influences both tumor cell survival and the immune consequences of radiotherapy, thereby affecting the therapeutic efficacy of radioimmunotherapy (Fig. 2; Table 2).

**Table 2:** Mechanistic bridges and therapeutic potential of E3 ubiquitin ligases in integrating radiation therapy and immune response modulation

Category	Name	Mechanism of Radio-Immunomodulation	Clinical Translation & Therapeutic Potential	References
DDR-Immune Sensing Bridge	RNF168	Stabilizes STAT1 via non-degradative ubiquitination, driving MHC-I and APM expression to convert “cold” tumors to “hot”.	Potential biomarker for radioimmunotherapy sensitivity.	[37, 38]
	SPOP	May regulate cytosolic DNA sensing pathways associated with cGAS-STING activation.	USP7 inhibitors or SPOP mimetics to restore TREX1 degradation.	[39–41]
	VHL-containing CRL complex	Regulates HIF-1α stability and hypoxia adaptation.	VHL-based PROTACs to eliminate oncogenic drivers while activating CTLs.	[42–44]
TME Remodeling & Evasion	ANAPC5	Facilitates radiation-induced CD24 membrane transport, sending “don’t eat me” signals to macrophages.	Targeted inhibition of the ANAPC5/GPAA1 axis to reverse immune evasion.	[45]
	TRIM21	Mediates VDAC2 degradation to sequester radiation-induced mtDNA, dampening innate immune sensing.	TRIM21 small-molecule inhibitors to enhance STING-mediated immunity.	[46]
	TRAF4	Drives K63-linked AKT ubiquitination to establish a dual defense of radioresistance and T-cell suppression.	Intervention against the TRAF4-AKT axis to overcome multi-modal resistance.	[49, 50]
Cell Death Patterning	MDM2	Attenuates RT effects via p53 degradation in tumors, but stabilizes STAT5 in T cells to sustain antitumor immunity.	MDM2-p53 antagonists (e.g., Alrizomadlin) to reverse resistance and trigger “in situ vaccine” effect.	[54–57]
	XIAP	Downregulation shifts radiation-induced death from “silent” apoptosis to ICD.	SMAC mimetics (e.g., Xevinapant) to trigger “in situ vaccine” effects.	[61–65]
	NEDD4L	Facilitates xCT ubiquitination to amplify radiation-induced ferroptosis and DC maturation.	Developing “radiotherapy-ferroptosis-immunotherapy” triple synergy.	[66–69]
	HACE1	Stabilizes NRF2 or degrades RAC1 to tune ROS levels; over-activation may block ICD and drive a “cold” phenotype, while moderate regulation may alleviate T-cell exhaustion.	Precise balance of oxidative equilibrium to achieve synergy between radiosensitization and immune activation.	[70–76]

### The interplay between DDR and immune sensing: From intranuclear signaling to antigen presentation

In addition to inducing DNA damage, radiation activates innate and adaptive immune signaling pathways. E3 ubiquitin ligases serve as a key bridge that translates DDR signals into signals recognizable by the immune system.

#### The RNF168-STAT1-MHC-I axis: mediating non-classical immunogenic conversion

The E3 ubiquitin ligase Ring finger protein 168 (RNF168) also exerts non-canonical immunoregulatory functions beyond DDR signaling. Notably, RNF168 does not regulate its substrate STAT1 through traditional degradation pathways; instead, it significantly enhances STAT1 protein stability by mediating non-degradative ubiquitination modifications (typically involving K63-linked chains or by antagonizing K48-linked chain formation), blocking its entry into the proteasomal degradation pathway [37, 38]. This enhanced stability enables STAT1 to efficiently translocate into the nucleus and activate the downstream transcription factor IRF1, which in turn directly drives the coordinated expression of MHC class I molecules and their core antigen processing machinery (APM, including the proteasome subunits LMP2/7 and the antigen transport protein TAP1) [37]. These findings suggest a potential link between RNF168-mediated STAT1 stabilization and enhanced antigen presentation capacity via the molecular hub RNF168. This mechanism provides a molecular basis for radiotherapy-induced enhancement of tumor immunogenicity and supports the rationale for targeting RNF168-mediated protein stability regulation to improve radioimmunotherapy efficacy.

#### The cGAS-STING pathway: Ubiquitination-driven regulation of the threshold for innate immune responses

Radiation therapy leads to the release of mitochondrial DNA and other cytoplasmic DNA, which is critical for activating the cGAS-STING pathway, inducing a type I interferon response, and promoting the maturation of dendritic cells (DCs). Speckle-type POZ protein (SPOP), the substrate-recognition subunit of the Cullin-3-like E3 ubiquitin ligase, acts as a critical rate-limiting factor determining the intensity of the aforementioned immune response by precisely regulating the proteostasis of the core nucleolytic enzyme TREX1. As the major cytoplasmic 3’-5’ DNA exonuclease in the body, TREX1 functions physiologically by efficiently clearing cytoplasmic DNA fragments to prevent excessive activation of autoimmune inflammation [39, 40]. However, in the tumor microenvironment, the abnormal overexpression of TREX1 constitutes a major obstacle to radiation-induced immune activation [41]. SPOP directly recognizes TREX1 and promotes its K48-linked polyubiquitination and degradation. This process is counteracted by the deubiquitinase USP7, and the balance between the two proteins determines cytoplasmic TREX1 abundance. Under radiation stress, SPOP-mediated TREX1 degradation significantly reduces the clearance rate of cytoplasmic dsDNA, lowering the molecular threshold for cGAS activation. This enables tumor cells to continuously secrete type I interferon (IFN-I) as well as chemokines such as CXCL10 and CCL5, enhancing the recruitment and activation of DCs and initiating an efficient systemic antitumor T-cell immune response. Experimental evidence indicates that functional defects in SPOP or overexpression of USP7 both lead to abnormal accumulation of TREX1, causing premature degradation of radiation-induced dsDNA before immune recognition, resulting in a “cold” tumor phenotype and resistance to radioimmunotherapy. Therefore, pharmacologically mimicking SPOP activity or applying USP7 inhibitors to restore TREX1 degradation potential has emerged as a cutting-edge translational strategy to enhance sensitivity to radioimmunotherapy and promote the transformation of “cold” tumors into “hot” tumors.

#### The VHL–HIF-1α axis: Coupling DDR pathway inhibition with hypoxic immune evasion

Tumor hypoxia is a common factor contributing to radiation resistance and immunosuppression. The functional subunit VHL (von Hippel-Lindau) of the Cullin-RING E3 ligase (CRL) plays a key role as a “molecular switch” in this process, integrating the mechanisms of radiosensitization and immune activation through a dual regulatory mechanism. On the one hand, VHL blocks the cascade activation of the DDR pathway by mediating the ubiquitination and degradation of oncogenic drivers (such as EGFR), significantly enhancing the sensitivity of tumor cells to ionizing radiation. This effect further triggers the ICD, enhances the capture and presentation of tumor-associated antigens by DCs, and consequently activates the specific killing activity of CD8⁺ cytotoxic T lymphocytes (CTLs) [42]. On the other hand, VHL precisely degrades hypoxia-inducible factor-1α (HIF-1α) through an oxygen-dependent pathway, reshaping the hypoxic microenvironment at the transcriptional level. This not only promotes the “normalization” of abnormal tumor vasculature by downregulating VEGF-A expression to improve oxygenation, but also effectively suppresses the chemotactic recruitment of myeloid-derived suppressor cells (MDSCs) and regulatory T cells (Tregs). Furthermore, VHL-mediated inactivation of HIF-1α synergistically reduces PD-L1 expression on the surface of tumor cells, reversing hypoxia-induced immune checkpoint evasion. The VHL–HIF-1α axis provides a profound mechanistic basis for overcoming tumor resistance to radiotherapy and immunotherapy by integrating a multidimensional network of “radiotherapy sensitization, vascular remodeling, and immune activation” [43, 44].

### Remodeling of the tumor microenvironment: Radiation-induced immunosuppression and escape mechanisms

Radiotherapy not only acts by directly killing tumor cells but also remodels the TME. However, the alterations in protein homeostasis induced by radiotherapy often trigger compensatory immune evasion. E3 ubiquitin ligases play a central role in radiotherapy-mediated immune tolerance by precisely regulating the stability of immune checkpoint proteins and related signaling pathways.

#### The ANAPC5/GPAA1 axis: Regulation of CD24 membrane transport and macrophage phagocytosis evasion

Recent findings highlight anaphase-promoting complex subunit 5 (ANAPC5) as a pivotal regulator in radiation-induced innate immune evasion. Ionizing radiation triggers the ANAPC5/GPAA1 axis, which drives the biosynthesis of GPI anchors and subsequently facilitates the translocation of CD24 to the cell surface. This enrichment of membrane-bound CD24 strengthens its engagement with Siglec-10 on macrophages, transmitting a potent ‘don’t eat me’ signal that inhibits phagocytic activity. By pharmacologically targeting the ANAPC5/GPAA1 signaling cascade, researchers can obstruct CD24 membrane expression and effectively sensitize tumors to radioimmunotherapy combinations [45].

#### TRIM21: A molecular switch mediating radiosensitivity and T-cell suppression

Tripartite Motif-containing Protein 21 (TRIM21), as a multifunctional E3 ubiquitin ligase, plays a key role in coordinating tumor radiosensitivity and TME remodeling. In a clinical cohort study of nasopharyngeal carcinoma (NPC), TRIM21 expression levels showed a significant negative correlation with patients’ radiotherapy outcomes and the degree of CD8⁺ T-cell infiltration within tumors, suggesting that it may serve as a central molecular switch for radiotherapy-induced immunosuppression. Mechanistically, TRIM21 orchestrates the K48-linked polyubiquitination and subsequent proteasomal degradation of VDAC2, sequestering radiation-induced mitochondrial DNA (mtDNA) within the mitochondria and impeding its cytosolic release. This molecular blockade ultimately dampens the innate immune response and diminishes antitumor immunity within the tumor microenvironment [46]. TRIM21 establishes a molecular barrier that protects tumor cells from radiation-induced antitumor immunity by regulating the “ubiquitination-VDAC2 degradation-mtDNA retention” axis. Therefore, the development of small-molecule inhibitors of TRIM21 or therapies targeting its ubiquitination pathway holds promise as a new strategy to enhance the efficacy of radioimmunotherapy for nasopharyngeal carcinoma.

#### The TRAF4-AKT axis: Cross-regulation of radioresistance and immune evasion

The AKT pathway is a central signaling cascade that regulates cell proliferation and survival, and its activity is precisely regulated by post-translational modifications mediated by E3 ubiquitin ligases. As a ring-finger domain E3 ligase of great interest in the field of oncology, TRAF4 drives the K63-linked ubiquitination and subsequent phosphorylation of AKT, establishing a dual defense mechanism of radiation resistance and immune evasion in various malignant tumors (such as OSCC, NPC, and CRC) [27, 47, 48]. In NPC, AKT activated by TRAF4 finely regulates CDK1 activity by phosphorylating Wee1 (Ser642) to maintain the cell cycle; simultaneously, it phosphorylates Survivin (Thr34) and, in conjunction with the TRAF4-mediated proteasome inhibition pathway, maintains the protein stability of Survivin, blocking the radiotherapy-induced apoptotic program [47, 48]. In Oral Squamous Cell Carcinoma (OSCC), IR can induce the interaction between TRAF4 and AKT, and mediate the K63-linked ubiquitination of AKT. The activated AKT inhibits the activity of GSK3β by phosphorylating its Ser9 site, which in turn reduces the phosphorylation of the anti-apoptotic protein MCL-1 at the S159 site. This process enhances the binding stability between MCL-1 and the deubiquitinase JOSD1, ultimately suppressing radiotherapy-induced cell apoptosis [47]. At a deeper biological level, TRAF4-mediated sustained AKT activation is a key driver of the remodeling of the TME. This uncontrolled signaling not only weakens the “in situ vaccine” effect of radiotherapy—which releases immunogenic factors—by enhancing intrinsic radiation resistance, but also creates a significantly immunosuppressive TME by promoting the functional activity of immunosuppressive cells (such as MDSCs and Tregs) and reducing the killing efficiency of effector T cells [49, 50]. TRAF4 bridges the gap between intrinsic cellular resistance and microenvironmental immunosuppression via the AKT pathway; its overexpression significantly attenuates the synergistic effects of radiotherapy and immune checkpoint inhibitor therapy. Targeted interventions against the TRAF4-AKT axis are expected to emerge as a new strategy for overcoming resistance to combined radiotherapy and immunotherapy.

### Intersections of cell death pathways: Remodeling cell death patterns to enhance immunogenicity

The immunogenic effects induced by radiation therapy are highly dependent on specific cell death mechanisms; precise manipulation of apoptosis, ferroptosis, and stress response pathways via E3 ubiquitin ligases can significantly enhance the release of damage-associated molecular patterns (DAMPs), facilitating the transition from “immunotolerant” to “immune-activating” cell death.

#### The MDM2-p53 axis: Coordinating tumor apoptosis and T-cell activity

As a central hub of the DDR, the p53 protein not only plays a critical role in cell cycle arrest and apoptosis but is also extensively involved in shaping the immune microenvironment by regulating the transcription of various chemokines and cytokines [51, 52]. In adenoid cystic carcinoma (ACC), approximately 5% of patients harbor TP53 gene mutations, which often portend a poor prognosis and treatment tolerance [53]. Studies have shown that the E3 ubiquitin ligase—murine double minute 2 (MDM2)—significantly attenuates the biological effects of radiation therapy by mediating the ubiquitination and degradation of p53 [54, 55]. Consequently, targeting the MDM2-p53 interaction has emerged as a highly promising strategy for radiosensitization. For example, clinical-stage MDM2 inhibitors (such as AMG 232) have been shown to effectively reverse radiation resistance in ACC [55]. Equally important are the non-classical functions of MDM2 in the context of immune regulation. In T cells, MDM2 exhibits protein homeostasis regulatory properties independent of p53: by competitively inhibiting STAT5 degradation mediated by the E3 ligase c-Cbl, it maintains the sustained activation of the STAT5 signaling pathway, enhancing the effector function of CD8⁺ T cells. This MDM2-mediated T-cell pathway is considered a key mechanism for optimizing the efficacy of immune checkpoint inhibitors (such as PD-L1 inhibitors) [56]. In addition, p53 can transcriptionally regulate downstream target genes (such as Bax and PUMA), causing radiation-induced cell death to exhibit stronger ICD characteristics (such as CRT exposure), exerting a synergistic immune-activating effect with MDM2 inhibitors [57].

#### XIAP: Reshaping the immunogenic profile of radiation-induced cell death

X-linked apoptosis inhibitory protein (XIAP), a core member of the inhibitory apoptosis protein (IAP) family, primarily antagonizes the activity of caspases-3, -7, and − 9 directly through its BIR domain and functions as an E3 ligase to degrade pro-apoptotic factors [58–60]. Radiotherapy often leads to limited immune activation due to the induction of “tolerant” apoptosis, whereas targeting XIAP can significantly alter the biological characteristics of cell death. When XIAP activity is downregulated or its function is inhibited using SMAC mimetics, the signaling pathways triggered by radiotherapy can shift from traditional “non-inflammatory” apoptosis to ICD [61]. This process induces the exposure of calreticulin on the cell surface, the release of ATP, and the extracellular secretion of high-mobility group box 1 (HMGB1). These molecular signals exert “find me” and “eat me” chemotactic effects, promoting DC recruitment, antigen uptake, and maturation, crossing the immune activation threshold [62, 63]. When radiotherapy is combined with XIAP-targeted intervention, tumor-derived antigens and DAMPs released locally can act as “in situ vaccines,” activating adaptive immunity. For example, AGuIX nanoparticles enhance radiotherapy-induced ICD, promote DC maturation and systemic T-cell responses, and demonstrate significant synergistic antitumor effects when combined with PD-1 blockade [64]. Similarly, nanoprobes enhance the abscopal effect of radiotherapy by alleviating intratumoral hypoxia and reducing the infiltration of immunosuppressive cells [63, 65]. XIAP inhibition enhances radiation-induced immunogenic cell death through caspase derepression, which subsequently promotes DAMP release and DC activation. This represents one of the key strategies for achieving the “radiation-induced in situ vaccine effect” and overcoming the bottleneck in radiotherapy-induced immune activation.

#### NEDD4L and ferroptosis: A new frontier in radiotherapy and chemotherapy combination therapy

Ferroptosis, a non-apoptotic, iron-dependent, lipid peroxidation-induced form of cell death, is emerging as a promising target for enhancing sensitivity to radiotherapy. Neural precursor cell expressed developmentally downregulated 4-like (NEDD4L), as a key E3 ubiquitin ligase, plays a central role in regulating the degradation of critical ferroptosis-related transporters, such as the amino acid transporter SLC7A11. Within the framework of combined radiotherapy and immunotherapy, radiotherapy induces lipid peroxidation by generating reactive oxygen species (ROS), while NEDD4L-mediated dysregulation of homeostasis further amplifies this effect, leading to widespread ferroptosis [66, 67]. Mechanistically, DAMPs released by ferroptotic cells can induce immature DCs to upregulate the expression of co-stimulatory molecules (such as CD80, CD86, and CD40), driving DC maturation. Mature DCs can more efficiently take up and present tumor antigens to T cells, successfully converting the local effects of radiotherapy into a systemic antitumor immune response [67]. Pharmacologically inducing ferroptosis not only overcomes resistance to conventional apoptosis but also, by reshaping the metabolic microenvironment, forms a potent synergy with immune checkpoint blockade (ICB), providing theoretical support for the development of a “radiotherapy-ferroptosis-immunotherapy” triple therapy [68, 69].

#### The HACE1/NRF2 axis: Bidirectional immune regulation of redox homeostasis

HECT domain and Ankyrin repeat-containing E3 ubiquitin-protein ligase 1 (HACE1) plays a key regulatory role in tumor radiation resistance and the shaping of the immune microenvironment by finely tuning cellular redox homeostasis. In the context of radiation therapy, IR induces ROS accumulation and tumor cell damage; however, tumor cells eliminate ROS by upregulating antioxidant mechanisms (such as the GSH-peroxidase system), developing radiation resistance [70]. Studies have shown that HACE1 enhances the stability of Nrf2 in gliomas by competitively binding to Keap1 and promoting its Ires-mediated translation [71]. Activated Nrf2 upregulates antioxidant genes and GSH levels, reducing radiotherapy-induced ROS accumulation and impairing radiosensitivity [72]. In sporadic Wilms tumor, HACE1 induces ubiquitin-dependent degradation of Ras-related C3 botulinum toxin substrate 1 (RAC1) [73], which reduces ROS production and attenuates radiotherapy efficacy [71]. When this regulatory role of oxidative stress extends to the immune microenvironment, it exhibits a high degree of context-dependence: On the one hand, HACE1 excessively suppresses ROS accumulation by activating the NRF2 pathway, potentially reducing tumor antigen release by blocking stress-induced ICD, driving a “cold tumor” phenotype, and leading to resistance to immunotherapy [74, 75]. On the other hand, given HACE1’s potential to mitigate inflammatory damage and improve the activation of MDSCs, moderate regulation of HACE1 may play a positive role in improving the immunosuppressive microenvironment by alleviating ROS-mediated T-cell exhaustion [76]. Therefore, intervention strategies targeting HACE1 must precisely balance its regulation of oxidative equilibrium to achieve synergy between radiosensitization and immune activation.

## Clinical translation: From molecular mechanisms to the precision oncology paradigm

Given the complex regulatory networks described above, E3 ubiquitin ligases not only constitute a critical juncture in fundamental biological mechanisms but have also evolved into an ideal intervention hub for clinically integrating radiotherapy, immunotherapy, and molecularly targeted therapy. By regulating substrate degradation or signal transduction mediated by ubiquitination, it is possible to achieve deep coupling of heterogeneous therapeutic strategies, providing a key breakthrough for precision combination therapy in oncology. This translational pathway follows a sequence from elucidating underlying mechanisms to validating target efficacy, and then, building upon the development of small-molecule lead compounds, drives the iterative optimization of radioimmunotherapy regimens, ultimately culminating in clinical trial evaluation and translation. These advances support the development of ubiquitination-guided multimodal therapeutic strategies in precision oncology.

### E3 ligase-based signatures for prognostic stratification and response prediction in radioimmunotherapy

The abnormal expression profiles of E3 ubiquitin ligases in tumor tissues are no longer viewed merely as byproducts of protein degradation dysregulation; instead, they have been shown to have deep causal links with the progression of malignant tumors and clinical outcomes. Through large-scale bioinformatics analysis and prospective clinical validation, specific E3 ubiquitin ligases have not only demonstrated precise potential for prognostic stratification but also play a decisive role in guiding personalized therapies (such as radiosensitivity and immune response).

Gene signatures constructed based on ubiquitination-related genes (URGs) have shown significant predictive value in clinical tumor assessment. In highly heterogeneous malignancies such as lung adenocarcinoma (LUAD), pancreatic ductal adenocarcinoma (PDAC), and ovarian cancer, risk scoring models integrating core URGs—including BTRC, CDB4, MDM2, and SKP2—enable precise patient stratification. Multiple validation studies based on the TCGA and GEO databases indicate that this model’s area under the curve (AUC) for predicting overall survival (OS) outperforms traditional TNM staging, and multivariate Cox regression analysis confirms it as an independent prognostic factor, independent of age, sex, and clinical stage [77–80].

Furthermore, the ubiquitination risk score effectively reflects the characteristics of the TME; the high-risk group is typically associated with reduced immune cell infiltration and differences in the expression of immune checkpoint molecules [79, 80]. This molecular subtyping based on the ubiquitination axis not only enhances the accuracy of prognostic assessment but also provides a scientific basis for identifying therapeutic targets and guiding personalized radiotherapy and immunotherapy. At the same time, E3 ubiquitin ligases offer unique advantages in predicting responses to radioimmunotherapy. Taking nasopharyngeal carcinoma as an example, the expression level of TRIM21 is negatively correlated with CD8⁺ T-cell infiltration, and this expression difference aids in the prospective identification of patient groups capable of generating a strong immune-sensitizing effect through activation of the cGAS-STING pathway following radiotherapy, laying a molecular foundation for the precise implementation of radioimmunotherapy [46, 81]. In summary, further exploration of the clinical value of E3 ubiquitin ligases holds significant academic and practical implications for improving the precision diagnosis and personalized treatment of tumors.

### Steering personalized integration of radiotherapy and immunotherapy

By quantitatively monitoring the expression profiles of specific E3 ubiquitin ligases, clinicians can move beyond traditional “pathological classification” to develop personalized combination treatment regimens based on proteostatic characteristics. Within the synergistic framework of radioimmunotherapy, E3 ubiquitin ligases play a dual role in regulating the release of DAMPs and the expression of immune checkpoints. In subgroups identified through clinical testing as having NPRL2 functional defects or FBXW7 inactivation, tumor cells—due to endogenous DNA repair defects and uncontrolled cell cycle progression—are highly susceptible to catastrophic genomic instability and micronucleus formation even under low-dose radiation exposure [82, 83]. This not only enhances radiation-induced cell death but also induces a robust interferon (IFN) response through activation of the cGAS-STING pathway. Such patients are considered “natural responders” to radioimmunotherapy and should receive conventional fractionated radiotherapy in combination with standard-dose ICIs. For MDM2-overexpressing radiation-resistant tumors, these often accelerate p53 degradation via ubiquitination pathways and interfere with the cGAS-STING-mediated interferon signaling pathway, attenuating radiation-induced immunogenicity release [84]. For such tumors, the strategy should be adjusted to employ high-dose, large-fraction radiotherapy (SBRT/hypofractionated RT) to forcibly trigger ICD through intense stress, combined with specific E3 ligase small-molecule inhibitors to block the degradation pathway and reverse immune evasion [85, 86].

The precise regulation of the PD-1/PD-L1 axis by E3 ubiquitin ligases directly determines whether radiotherapy-induced antitumor immunity can be sustained. When a patient’s expression profile indicates low activity levels of E3 ubiquitin ligases (such as SPOP) that mediate PD-L1 degradation, the PD-L1 upregulated by radiotherapy cannot be effectively cleared, leading to rapid exhaustion of the T-cell infiltration induced by radiotherapy [87]. To address this molecular phenotype, clinical strategies should evolve from simple combination therapy to a “triple sequential strategy”: specifically, building upon radiotherapy, introducing a targeted E3 ligase-targeted proteolytic complex to restore the degradation homeostasis of PD-L1, and synergizing with anti-PD-1 monoclonal antibodies to enhance immune remodeling in “cold tumors.” Through this precision selection based on E3 expression profiles, clinical decision-making can accurately identify which patients require only conventional radiotherapy and immunotherapy, and which patients must be supplemented with E3 modulators to overcome immune tolerance. This approach maximizes the synergistic effects of radiotherapy and immunotherapy while avoiding unnecessary systemic toxicity.

### Dissecting the molecular mechanisms underlying multimodal treatment of drug resistance

In clinical practice involving radioimmunotherapy, although the two modalities have synergistic potential, most patients ultimately experience disease progression due to primary or acquired resistance. This resistance does not stem from the failure of a single pathway but is driven by E3 ubiquitin ligase-mediated remodeling of protein homeostasis. Under the dual selection pressures of radiation and immune response, tumor cells establish a complex defense mechanism to evade cell death and immune clearance by precisely regulating the degradation kinetics of specific proteins. The failure of multimodal therapy is often accompanied by the evolution of the TME toward an immunosuppressive phenotype. E3 ubiquitin ligases play a molecular anchoring role in maintaining this “cold” phenotype. Loss-of-function mutations in SPOP or COP1 overexpression are frequently detected in clinically resistant samples. As a negative regulator of PD-L1, the loss of SPOP leads to non-transcriptionally dependent accumulation of PD-L1 at the protein level. During radioimmunotherapy, IFN-γ upregulated by radiation therapy would normally induce an increase in PD-L1. However, if the degradation mechanism fails, the sustained high expression of PD-L1 will rapidly drive newly infiltrating CD8⁺ T cells into an irreversible state of exhaustion, directly blocking the subsequent amplifying effects of ICIs [88, 89]. In addition to immune checkpoint evasion, defects in the E3 ubiquitin ligase-mediated antigen presentation mechanism are a key factor contributing to radiation resistance in patients. The “in situ vaccine” effect induced by radiation therapy is highly dependent on the presentation of tumor antigens by MHC-I molecules. In addition to its classical receptor function, PD-L1 possesses atypical E3 ligase activity, capable of directly ubiquitinating β2-microglobulin and inducing its degradation, thereby fundamentally disrupting the structural integrity and surface expression of MHC-I [90]. Furthermore, WWP2—identified through genome-wide CRISPR screening—along with members of the TRIM and MARCH families under specific microenvironmental conditions, constitute a complex network of negative regulation of MHC-I [91]. Therefore, interventions targeting these specific ubiquitination nodes—such as restoring SPOP function, inhibiting WWP2 activity, or disrupting the non-canonical enzymatic activity of PD-L1—not only reconstruct the antigen recognition cascade but also hold promise for reversing the “cold tumor” phenotype, providing precise translational medical strategies to overcome clinical drug resistance.

### The leap of therapeutic paradigms from classical pharmacological modulation to induced protein degradation

Recent advances in ubiquitin biology have facilitated the development of therapeutic strategies targeting protein homeostasis in radioimmunotherapy. Several studies are evaluating molecules such as RNF168, STAT1, and cGAS as potential biomarkers for predicting treatment response. At the therapeutic level, current approaches mainly focus on two directions: pharmacological modulation of E3 ligase activity using small-molecule inhibitors or agonists, and targeted protein degradation technologies including PROTACs and MGDs. These approaches aim to enhance radiosensitivity, restore antitumor immune activity, and overcome resistance associated with aberrant ubiquitin signaling (Table 3).

**Table 3:** Current landscape of clinical translation: Drug candidates targeting the ubiquitin-proteasome system for multimodal cancer therapy

Category	Drug Candidate	Target	Mechanism of Action	Clinical Trials (Status)	Target Indications	References
IAP Inhibitors	Xevinapant (Debio 1143)	cIAP1/2, XIAP	Bivalent SMAC mimetic; induces cIAP auto-ubiquitination	NCT02022098 (Ph II) TrilynX NCT04459715 (Ph III)	LA-SCCHN	[92–96]
	Tolinapant (ASTX660)	cIAP1/2, XIAP	High-affinity non-peptide IAP antagonist	NCT05245682 (Ph I)	HNSCC Solid tumors	[97]
	LCL161	cIAP1/2	Activates non-classical NF-κB pathway via cIAP degradation	NCT01098838 (Ph I) NCT02649673 (Ph Ib)	SCLC Gynecologic cancers	[98, 99]
MDM2-p53 Antagonists	Alrizomadlin (APG-115)	MDM2/p53	Blocks MDM2-p53 interaction; restores p53-mediated apoptosis	NCT02935907 (Ph I) NCT03611868 (Ph II)	Melanoma DDLPS	[100–102]
	Siremadlin (HDM201) Idasanutlin (RG7388)	MDM2	Highly selective MDM2 inhibitor; induces PUMA/BAX	NCT03032523 (Ph III)	AML Solid tumors	[104–107]
DNA Damage Repair Inhibitors	Peposertib (M3814)	DNA-PKcs	Inhibits DNA-PK-mediated NHEJ repair	NCT03724890 (Ph II)	Advanced solid tumors	[108–110]
PROTACs	Vepdegestrant (ARV-471)	ER (VHL-recruiting)	Induces ER ubiquitination and proteasomal degradation	NCT05654623 (Ph III)	ER+/HER2- breast cancer	[116, 117]
	Bavdegalutamide (ARV-110)	AR (CRBN-recruiting)	Degrades full-length and mutant AR (T878X/H875Y)	NCT03888612 (Ph I/II)	mCRPC	[118]
	KT-333	STAT3	First-in-class STAT3 degrader; eliminates scaffolding functions	NCT05225584 (Ph I)	Lymphoma Solid tumors	[120]
Molecular Glues	Mezigdomide (CC-92480)	CRBN/IKZF1-3	Next-gen CELMoD; triggers T-cell IFN pathway activation	NCT03374085 (Ph I/II) NCT05552976 (Ph III)	Refractory Multiple Myeloma	[123]

#### Inhibitors

E3 ubiquitin ligase inhibitors exert antitumor effects by binding to the catalytic domains of the enzymes, blocking their interactions with target proteins, inhibiting ubiquitination function, and thereby stabilizing tumor suppressor proteins or degrading oncoproteins. IAP antagonists represent one of the most extensively studied classes. As the most rapidly advancing bivalent SMAC mimetic in this field, Xevinapant (Debio 1143) induces the auto-ubiquitination and degradation of cIAP1/2 by mimicking the binding of endogenous SMAC proteins to IAPs, releasing the inhibition of caspase activity and altering the immune microenvironment [92, 93]. Clinical evidence has preliminarily confirmed the significant benefits of combining IAP inhibitors with radiotherapy. In a randomized Phase II clinical trial (NCT02022098), Xevinapant combined with standard chemoradiotherapy significantly improved patients’ 3-year progression-free survival (PFS) and overall survival (OS), and 5-year follow-up data further validated the sustained survival benefit [94, 95]. Based on these findings, the large-scale, international, multicenter Phase III TrilynX trial (NCT04459715) is currently evaluating the efficacy of Xevinapant in combination with chemoradiotherapy in patients with unresectable locally advanced (LA) SCCHN (oropharyngeal, hypopharyngeal, and laryngeal) [96].

Another high-affinity non-peptide IAP antagonist, Tolinapant (ASTX660), is also actively being explored in combination with radiotherapy. An open-label, single-arm Phase I trial (NCT05245682) evaluated the safety and feasibility of Tolinapant in combination with definitive radiation therapy in patients with locally advanced HNSCC who were intolerant to cisplatin. At a median follow-up of 13.8 months, 70% of patients were progression-free [97].

In addition to the aforementioned bivalent SMAC mimetics, LCL161, a monovalent IAP antagonist developed by Novartis, has also demonstrated unique therapeutic potential in clinical studies. Similar to Xevinapant, LCL161 activates the non-classical NF-κB signaling pathway by inducing the degradation of cIAP1/2, triggering tumor necrosis factor (TNF)-mediated apoptosis. A Phase I clinical trial (NCT01098838) demonstrated that LCL161 monotherapy exhibited good safety in patients with advanced solid tumors, with disease stabilization observed in some cases [98]. Given the limited efficacy of monotherapy, multi-phase clinical trials are currently underway to evaluate LCL161 in combination with other anticancer drugs, including topotecan, for the treatment of recurrent/refractory small cell lung cancer and certain gynecological malignancies (NCT02649673), with the aim of enhancing therapeutic outcomes through synergistic effects [99].

From a mechanistic perspective, IAP inhibitors not only directly trigger ICD through E3 ligase-mediated degradation, but also synergize with the radiation-induced “in situ vaccine” effect. By enhancing activation of the cGAS-STING pathway and improving T-cell infiltration, they overcome acquired resistance to immune checkpoint blockade therapy. The advancement of these clinical trials marks a transition for E3 ligase-targeted drugs from a single-dimensional “cytotoxicity sensitization” approach to a new era of precision radiation-immunotherapy focused on “regulatory control of the immune microenvironment.”

MDM2 inhibitors represent another important category of inhibitors. MDM2 can bind to and ubiquitinate p53 for degradation, inhibiting its tumor-suppressive function, and is highly expressed in p53-wild-type tumors. This class of inhibitors competitively blocks the MDM2-p53 interaction by mimicking the N-terminal domain of p53, suppresses the E3 ubiquitin ligase activity of MDM2, stabilizes and activates p53, and induces cell cycle arrest and apoptosis in tumor cells. Currently, APG-115 (Alrizomadlin), a rapidly advancing MDM2-p53 antagonist, has demonstrated significant therapeutic potential in multiple clinical studies. In a pioneering first-in-human Phase I dose-escalation and expansion study (NCT02935907), Alrizomadlin demonstrated a manageable safety profile, with the maximum tolerated dose (MTD) and recommended Phase II dose (RP2D) determined to be 150 mg and 100 mg, respectively. Notably, in patients with MDM2 amplification and wild-type TP53, particularly in the dedifferentiated liposarcoma (DDLPS) subgroup, Alrizomadlin demonstrated robust preliminary clinical efficacy, highlighting the therapeutic potential of precision p53 restoration therapy [100]. In a study of solid tumors resistant to chemoradiotherapy, results from a Phase II clinical trial (NCT03611868) indicated that the combination of Alrizomadlin and Pembrolizumab demonstrated significant clinical activity in patients with highly refractory, unresectable, or metastatic cutaneous melanoma who had previously failed treatment with PD-1/PD-L1 inhibitors. This finding confirms that stabilizing wild-type p53 through pharmacological intervention not only induces an “in situ vaccine effect” in tumors but also clinically validates its critical role in reshaping the immune microenvironment, reversing ICB resistance, and synergistically enhancing the radioimmunological response [100–102].

Meanwhile, Milademetan (DS-3032), developed by Daiichi Sankyo, has also been shown in preclinical and early clinical studies to enhance radiation therapy-induced type I interferon responses by upregulating the cGAS-STING pathway; this mechanism holds unique promise for the radiosensitization of DDLPS [103].

In the field of highly selective small-molecule inhibitors, Siremadlin (HDM201), developed by Novartis, and Idasanutlin (RG7388), developed by Roche, have become the focus of research in precision medicine. Clinical evidence shows that both compounds have demonstrated significant radiotherapy-sensitizing potential in Phase I/II clinical evaluations for acute myeloid leukemia (AML) and various solid tumors, particularly in synergistically inducing the upregulation of pro-apoptotic factors such as PUMA and BAX following combined radiation therapy [104–107]. Although early Phase I/II data were promising, the Phase III MIRROS trial (NCT02545283) failed to meet its primary endpoint in TP53-WT patients. The heterogeneity in the efficacy of idasanutlin observed in late-stage clinical studies suggests that the clinical application of MDM2 inhibitors is highly dependent on precise molecular screening for TP53 status and in-depth identification of accompanying mutations (such as MDM2 amplification and ATM deletion). Future research will focus on optimizing dosing sequences and adopting intermittent dosing strategies to mitigate hematologic toxicity, as well as developing more rigorous multi-marker biomarker systems. These efforts aim to overcome acquired resistance in complex tumor microenvironments and maximize the clinical value of MDM2 inhibitors in combination with radioimmunotherapy.

Peposertib (M3814) is a selective inhibitor of DNA-dependent protein kinase (DNA-PKcs), a key enzyme in the non-homologous end joining (NHEJ) pathway of DNA double-strand break repair. By inhibiting DNA-PKcs, Peposertib can enhance tumor cell radiosensitivity, increasing the persistence of radiation-induced DNA damage. In a Phase II clinical trial (NCT03724890), Peposertib administered alone or in combination with Avelumab and radiotherapy was well tolerated in patients with advanced solid tumors. Preclinical studies suggest that DNA-PK inhibition by Peposertib may also promote immunogenic cell death and enhance antitumor immune responses, providing a rationale for potential combination strategies with immune checkpoint blockade [108–110].

#### Agonists

E3 ligase agonists exert antitumor effects by enhancing the activity or stability of E3 ubiquitin ligases, thus promoting the ubiquitination and degradation of oncoproteins. However, their development poses greater challenges than that of inhibitors, and they are still in the early stages of research. For instance, agonists targeting SCFβ-TrCP can enhance the degradation of oncoproteins such as β-catenin and cyclin E, inhibit the Wnt/β-catenin pathway, and thereby suppress the growth of tumors, including colorectal cancer and liver cancer [111]. Natural products like curcumin derivatives can activate the E3 ligase RNF41, facilitate the degradation of HER2, and enhance the sensitivity of tumor cells to targeted drugs. Currently, the research focus of such agents lies in target validation and small-molecule compound screening, while their clinical translation still faces challenges such as low specificity and poor bioavailability [112].

#### PROTACs

PROTACs are a class of bifunctional small molecules composed of a target protein ligand, an E3 ubiquitin ligase-recruiting ligand, and a linker. They transcend the limitations of traditional inhibitors, serving not only as highly efficient protein degraders but also playing a key role as “radiation response modulators” in the field of radiobiology. By eliminating the scaffolding functions of oncogenic proteins that are difficult to target with conventional drugs, PROTACs can eradicate the root causes of drug resistance at the proteomic level, opening up a new strategic dimension for overcoming radiation resistance [113].

To date, research on PROTACs has extensively covered a wide range of tumor-associated target proteins, with studies on proteins of the bromo- and external-domain (BET) family being particularly in-depth. As the first reported BET-targeting PROTAC, MZ1 induces efficient ubiquitination and degradation of BRD4 by specifically recruiting the VHL E3 ubiquitin ligase [114]. Since BRD4 recognizes acetylated histones and drives the expression of oncogenes such as c-Myc and BCL-2, it is highly expressed in hematological malignancies such as acute myeloid leukemia (AML). Studies have shown that MZ1 exhibits significant antitumor activity in AML models; by eliminating BRD4, it effectively inhibits the c-Myc signaling pathway and induces apoptosis, validating the potential of this technology in epigenetic regulation [115].

In the field of clinical translation, degraders targeting the androgen receptor (AR) and estrogen receptor (ER) are already at the forefront. Vepdegestrant (ARV-471) is an oral PROTAC targeting the ER and is currently in Phase III clinical trials (NCT05654623). Recent clinical data show that in patients with ER+/HER2- advanced breast cancer, vepdegestrant demonstrated superior progression-free survival (PFS) compared to the standard therapy fulvestrant in the ER1-mutated subgroup, highlighting its unique advantage in overcoming receptor mutation-driven resistance [116, 117]. Meanwhile, Bavdegalutamide (ARV-110), the first AR degrader to enter clinical trials, has demonstrated highly promising efficacy in metastatic castration-resistant prostate cancer (mCRPC). A Phase I/II clinical study (NCT03888612) showed that ARV-110 demonstrated good safety in a cohort of severely ill patients who had received a median of 4 prior lines of therapy, and established the RP2D as 420 mg QD. Its efficacy is significantly biomarker-dependent: in patients harboring AR T878X or H875Y mutations, the PSA50 response rate reached 46%. Since such ligand-binding domain (LBD) mutations often lead to acquired resistance to abiraterone or enzalutamide, ARV-110 successfully blocks the activation of escape signaling pathways by completely degrading both the full-length receptor and its mutant forms. Based on these breakthroughs, AR degraders are laying a new theoretical foundation for combined radiotherapy and immunotherapy. Unlike the competitive inhibition of traditional antagonists, PROTAC-mediated degradation more thoroughly weakens the AR’s transcriptional regulation of DDR genes, thereby producing a potent radiosensitizing effect. The current clinical trend has shifted toward exploring the combination of degraders with radioligand therapy or ICIs, aiming to synergistically enhance antitumor immune responses and broaden the therapeutic window by clearing receptor splice variants and mutants [118].

The novel degrader DT-2216 (which targets BCL-XL) demonstrates exceptional clinical potential for radiosensitization and DNA damage repair. Compared to the traditional inhibitor Navitoclax, DT-2216 leverages the low expression of VHL E3 ligase in platelets to significantly reduce platelet toxicity while precisely inducing apoptosis in radiation-induced senescent tumor cells, demonstrating a significant ‘radiation-proteolysis’ synergistic effect [119].

As the first-in-class STAT3 degrader, the development of KT-333 addresses the long-standing challenge of disrupting the non-transcriptional scaffolding function of STAT3. Unlike traditional SH2 domain inhibitors, which often trigger compensatory feedback, KT-333 promotes near-complete proteasomal clearance of STAT3 (with a degradation rate as high as 97.5% in clinical peripheral blood mononuclear cell samples, NCT05225584). This systemic depletion not only directly inhibits tumor proliferation signals but also fundamentally reshapes the immune microenvironment. By eliminating STAT3-mediated inhibitory signals in MDSCs and enhancing interferon-γ gene expression, KT-333 acts as a catalyst when used in combination with radiation therapy, generating a potent “in situ vaccine” effect [120]. These clinically stage-ready degraders highlight a key shift in radiopharmaceutical strategies: leveraging TPD to simultaneously enhance radiosensitivity and dismantle the immune evasion barriers established by therapeutic stress.

The clinical application of PROTACs continues to face multiple challenges, such as poor membrane permeability due to high molecular weight, the impact of linker design on degradation efficiency, toxicity resulting from off-target degradation, and high costs associated with complex manufacturing processes. Current research is primarily focused on optimizing molecular structures, improving bioavailability, and enhancing tissue specificity.

#### MGDs

MGDs are small-molecule compounds with simple structures, and their mechanism of action differs from that of PROTACs. Instead of requiring the design of a target protein-binding domain, MGDs induce the formation of novel protein-protein interactions (PPIs) between E3 ubiquitin ligases and target proteins, thereby enabling the ubiquitination and degradation of target proteins that were not originally recognized by E3 ubiquitin ligases [121]. Thalidomide and its analogs (lenalidomide, pomalidomide) are the most representative MGDs. These compounds can bind to the E3 ubiquitin ligase cereblon, induce its conformational changes, promote its recognition and binding to novel target proteins such as IKZF1, IKZF3, and CK1α, and further mediate the degradation of these target proteins. For example, in multiple myeloma (MM) cells, lenalidomide can induce the binding of CRBN to IKZF1/3. The degradation of these two proteins activates the expression of CD20 downstream of IRF4, enhances the sensitivity of MM cells to rituximab, and simultaneously inhibits c-Myc expression to exert antitumor effects [122].

In addition to the thalidomide analogs mentioned above, the discovery of novel MGDs is reshaping the landscape of cancer immunotherapy. Phase I/II clinical trials (NCT03374085) and the latest Phase III SUCCESSOR-1 study (NCT05552976) have confirmed that Mezigdomide (CC-92480), as a next-generation CRBN modulator, demonstrates potent antitumor activity in patients resistant to lenalidomide/ pomalidomide, not only directly inducing plasma cell apoptosis but also synergizing with the in situ vaccine effect generated by radiation through activation of the T-cell interferon pathway, significantly enhancing the immunogenicity of solid tumors [123].

In non-CRBN-dependent pathways, Indisulam (E7070), a representative MGD that targets DCAF15, induces the degradation of the splicing factor RBM39. This degradation leads to widespread RNA splicing errors within tumor cells, generating a large number of neoantigens, further enhancing the antitumor response of CD8⁺ T cells in the radiation-induced microenvironment [124, 125].

MGDs have certain limitations, including unpredictable target protein profiles, severe adverse reactions associated with some compounds (e.g., the teratogenicity of thalidomide), and the high tendency of tumor cells to develop drug resistance through E3 ligase mutations or downregulated target protein expression. Future research and development priorities will focus on developing highly specific MGDs via high-throughput screening technologies, elucidating the structures of MGD-mediated PPIs, and optimizing compound structures to reduce toxicity.

## E3 ubiquitin ligases: A central molecular hub for integrating cancer therapy

E3 ubiquitin ligases participate in multiple biological processes relevant to radiotherapy and tumor immunity, including DNA damage repair, immune checkpoint regulation, redox homeostasis, and cell death signaling. Increasing evidence indicates that ubiquitin-dependent pathways influence not only intrinsic tumor radiosensitivity but also radiation-induced immune activation within the tumor microenvironment. Because these ligases regulate both stress adaptation and antitumor immunity, they have emerged as potential therapeutic targets for combination strategies involving radiotherapy and immunotherapy.

From the perspective of radiation therapy, E3 ubiquitin ligases (such as RNF168 and TRIP12) serve as early sensors of the DDR. By recruiting repair factors to sites of damage, they not only determine the radiosensitivity of tumor cells but also convert physical damage signals into immune activation signals by regulating intrinsic innate immune sensors such as the cGAS-STING pathway. This ability to mediate cross-domain regulation between radiotherapy and immunotherapy establishes the theoretical basis for E3 ubiquitin ligases as synergistic agents in combined radiotherapy and immunotherapy.

In shaping the immune microenvironment, E3 ubiquitin ligases act as “immune sentinels.” They not only directly control the spatiotemporal stability of immune checkpoints such as PD-L1 and CTLA-4 but also deeply participate in the turnover of antigen-processing and presentation-related proteins (such as MHC-I). This regulatory mechanism endows E3 ubiquitin ligases with the potential to reshape the tumor immune phenotype (from “cold” to “hot”), providing a new logical starting point for overcoming resistance to ICIs.

As integrative hubs, the clinical translational value of E3 ubiquitin ligases has been unleashed to an unprecedented extent through protein degradation technologies such as PROTACs and molecular glues. Unlike traditional small-molecule inhibitors, E3 ligase-based degradation strategies have achieved a paradigm shift from “functional inhibition” to “protein clearance.” By designing bifunctional molecules, researchers can leverage E3 ubiquitin ligases that are tumor-specifically expressed or radiation-induced to precisely degrade key oncoproteins driving tumor progression and immune suppression. This strategy not only overcomes kinase mutation resistance in traditional targeted therapies but also further enhances systemic immune responses by inducing ICD.

E3 ubiquitin ligases have transcended the realm of basic biology to become a central hub for interdisciplinary therapeutic strategies. Future challenges lie in elucidating the “heterogeneous functions” of E3 ubiquitin ligases under different therapeutic pressures and utilizing chemical biology tools to precisely manipulate this molecular node, achieving the deep integration of radiation therapy, immune activation, and protein degradation therapies and driving clinical breakthroughs.

## Future prospects

Although the central role of E3 ubiquitin ligases in regulating sensitivity to radioimmunotherapy has been preliminarily confirmed, their translation into routine clinical diagnostic and therapeutic protocols remains in its infancy. Future research should first focus on developing a precision biomarker system based on proteome-wide stability profiles. By leveraging single-cell proteomics and spatial transcriptomics, researchers can dynamically define the specific “ubiquitination fingerprints” of tumor cells and the immune microenvironment under radiation stress, thereby identifying patient subgroups likely to benefit from radioimmunotherapy. In terms of drug design, to address the toxicity and resistance issues commonly faced by current small-molecule inhibitors, efforts should be directed toward developing a new generation of degradation strategies with spatiotemporal controllability, such as photosensitive PROTACs or hypoxia-inducible molecular glues. This would enable protein degradation to be activated only within the radiation-irradiated region, maximizing local radioimmunotherapy synergy while avoiding systemic immunotoxicity. Furthermore, the application of synthetic lethal strategies holds great promise. By precisely screening for E3 ligase targets that are complementary to DNA damage repair defects (such as ATM/BRCA mutations), it is expected to induce strong activation of the cGAS-STING pathway under low-dose radiation, generating a significant “distant effect.” Finally, there is an urgent need to integrate genome-wide CRISPR screening with artificial intelligence algorithms to systematically identify novel E3-substrate interaction interfaces that mediate immune evasion, and to deeply couple ubiquitination regulation with immune checkpoint blockade (ICB) and novel cell death pathways such as ferroptosis. This paradigm shift from targeting single nodes to systemic proteomic remodeling will provide a novel clinical translation pathway for overcoming radiation-resistant tumors.

## Data Availability

No datasets were generated or analysed during the current study.

## Abbreviations

DDR: DNA damage response
TME: Tumor microenvironment
ICD: Immunogenic cell death
PROTACs: Proteolysis-targeting chimeras
MGDs: Molecular glue degraders
UPS: Ubiquitin-proteasome system
HECT: Homologous to the c-terminus of the e6-associated protein
RING: Really interesting new gene
RBR: RING-between-RING
IR: Ionizing radiation
DSBs: DNA double-strand breaks
HR: Homologous recombination
NHEJ: Non-homologous end joining
MRN: MRE11/RAD50/NBS1
cIAP2: Cellular inhibitor of apoptosis 2
TNBC: Triple-negative breast cancer
RNF126: Ring finger protein 126
ESCC: Esophageal squamous cell carcinoma
BC: Breast cancer
UHRF1: Ubiquitin-like with PHD and ring finger domains 1
RNF8: Ring finger protein 8
RNF216: Ring finger protein 216
GBM: Glioblastoma multiforme
CRC: Colorectal Cancer
HDR: Homology-directed repair
TRAF4: TNF Receptor Associated Factor 4
ICIs: Immune checkpoint inhibitors
NEDD4: Neural precursor cell expressed developmentally downregulated 4
FGFR3: Fibroblast growth factor receptor 3
Cbl-b: Casitas B-lineage lymphoma-b
TRIM71: Tripartite motif-containing protein 71
PLC: Peptide loading complex
COP1: Component of photomorphogenic complex 1
ICB: Immune checkpoint blockade
RNF126: Ring finger protein 126
EMT: Epithelial-mesenchymal transition
DCs: Dendritic cells
SPOP: Speckle-type POZ protein
IFN-I: Type I interferon
CRL: Cullin-RING E3 ligase
CTLs: CD8⁺ cytotoxic T lymphocytes
HIF-1α: Hypoxia-inducible factor-1α
MDSCs: Myeloid-derived suppressor cells
Tregs: Regulatory T cells
ANAPC5: Anaphase-promoting complex subunit 5
TRIM21: Tripartite Motif-containing Protein 21
NPC: Nasopharyngeal carcinoma
mtDNA: Mitochondrial DNA
OSCC: Oral squamous cell carcinoma
DAMPs: Damage-associated molecular patterns
ACC: Adenoid cortical carcinoma
MDM2: Murine double minute 2
XIAP: X-linked apoptosis inhibitory protein
IAP: Inhibitory apoptosis protein
HMGB1: High-mobility group box 1
NEDD4L: Neural precursor cell expressed developmentally downregulated 4-like
ROS: Reactive oxygen species
ICB: Immune checkpoint blockade
HACE1: HECT domain and Ankyrin repeat-containing E3 ubiquitin-protein ligase 1
URGs: Ubiquitination-related genes
LUAD: Lung adenocarcinoma
PDAC: Pancreatic ductal adenocarcinoma
AUC: Area under the curve
OS: Overall survival
IFN: Interferon
DDLPS: Dedifferentiated liposarcoma
AML: Acute myeloid leukemia
AR: Androgen receptor
ER: Estrogen receptor
PFS: Progression-free survival
mCRPC: Metastatic castration-resistant prostate cancer
LBD: Ligand-binding domain

## References

[1] Huang R-X, Zhou P-K. DNA damage response signaling pathways and targets for radiotherapy sensitization in cancer. Signal Transduct Target Ther. 2020;5:60. 10.1038/s41392-020-0150-x. 32355263 PMC7192953

[2] Zheng S, Wu Y, Li Z. Integrating cullin2-RING E3 ligase as a potential biomarker for glioblastoma multiforme prognosis and radiosensitivity profiling. Radiother Oncol J Eur Soc Ther Radiol Oncol. 2021;154:36–44. 10.1016/j.radonc.2020.09.005. 32918970

[3] Riley RS, June CH, Langer R, Mitchell MJ. Delivery technologies for cancer immunotherapy. Nat Rev Drug Discov. 2019;18:175–96. 10.1038/s41573-018-0006-z. 30622344 PMC6410566

[4] Schreiber RD, Old LJ, Smyth MJ. Cancer immunoediting: integrating immunity’s roles in cancer suppression and promotion. Science. 2011;331:1565–70. 10.1126/science.1203486. 21436444

[5] Seo Y-S, Ko IO, Park H, Jeong YJ, Park J-A, Kim KS, et al. Radiation-Induced Changes in Tumor Vessels and Microenvironment Contribute to Therapeutic Resistance in Glioblastoma. Front Oncol. 2019;9:1259. 10.3389/fonc.2019.01259. 31803626 PMC6873882

[6] Maity A, McKenna WG, Muschel RJ. The molecular basis for cell cycle delays following ionizing radiation: a review. Radiother Oncol J Eur Soc Ther Radiol Oncol. 1994;31:1–13. 10.1016/0167-8140(94)90408-1. 8041894

[7] Igney FH, Krammer PH. Death and anti-death: tumour resistance to apoptosis. Nat Rev Cancer. 2002;2:277–88. 10.1038/nrc776. 12001989

[8] Lynam-Lennon N, Reynolds JV, Pidgeon GP, Lysaght J, Marignol L, Maher SG. Alterations in DNA repair efficiency are involved in the radioresistance of esophageal adenocarcinoma. Radiat Res. 2010;174:703–11. 10.1667/RR2295.1. 21128793

[9] Rui R, Zhou L, He S. Cancer immunotherapies: advances and bottlenecks. Front Immunol. 2023;14:1212476. 10.3389/fimmu.2023.1212476. 37691932 PMC10484345

[10] Hoeller D, Hecker C-M, Dikic I. Ubiquitin and ubiquitin-like proteins in cancer pathogenesis. Nat Rev Cancer. 2006;6:776–88. 10.1038/nrc1994. 16990855

[11] Buetow L, Huang DT. Structural insights into the catalysis and regulation of E3 ubiquitin ligases. Nat Rev Mol Cell Biol. 2016;17:626–42. 10.1038/nrm.2016.91. 27485899 PMC6211636

[12] Wang H, Lu Y, Wang M, Wu Y, Wang X, Li Y. Roles of E3 ubiquitin ligases in gastric cancer carcinogenesis and their effects on cisplatin resistance. J Mol Med Berl Ger. 2021;99:193–212. 10.1007/s00109-020-02015-5. 33392633

[13] Zhang M, Shao Y, Gu W. The Mechanism of Ubiquitination or Deubiquitination Modifications in Regulating Solid Tumor Radiosensitivity. Biomedicines. 2023;11:3240. 10.3390/biomedicines11123240. 38137461 PMC10741492

[14] Tan J, Sun X, Zhao H, Guan H, Gao S, Zhou P-K. Double-strand DNA break repair: molecular mechanisms and therapeutic targets. MedComm. 2023;4:e388. 10.1002/mco2.388. 37808268 PMC10556206

[15] Shibata A, Moiani D, Arvai AS, Perry J, Harding SM, Genois M-M, et al. DNA double-strand break repair pathway choice is directed by distinct MRE11 nuclease activities. Mol Cell. 2014;53:7–18. 10.1016/j.molcel.2013.11.003. 24316220 PMC3909494

[16] Nicholson J, Jevons SJ, Groselj B, Ellermann S, Konietzny R, Kerr M, et al. E3 Ligase cIAP2 Mediates Downregulation of MRE11 and Radiosensitization in Response to HDAC Inhibition in Bladder Cancer. Cancer Res. 2017;77:3027–39. 10.1158/0008-5472.CAN-16-3232. 28363998

[17] Liu W, Zheng M, Zhang R, Jiang Q, Du G, Wu Y, et al. RNF126-Mediated MRE11 Ubiquitination Activates the DNA Damage Response and Confers Resistance of Triple-Negative Breast Cancer to Radiotherapy. Adv Sci Weinh Baden-Wurtt Ger. 2023;10:e2203884. 10.1002/advs.202203884. PMC9929257 36563124

[18] Wang Q, Goldstein M, Alexander P, Wakeman TP, Sun T, Feng J, et al. Rad17 recruits the MRE11-RAD50-NBS1 complex to regulate the cellular response to DNA double-strand breaks. EMBO J. 2014;33:862–77. 10.1002/embj.201386064. 24534091 PMC4194111

[19] Groselj B, Kerr M, Kiltie AE. Radiosensitisation of bladder cancer cells by panobinostat is modulated by Ku80 expression. Radiother Oncol J Eur Soc Ther Radiol Oncol. 2013;108:429–33. 10.1016/j.radonc.2013.06.021. PMC3824066 23932191

[20] Li X, Meng Q, Rosen EM, Fan S. UHRF1 confers radioresistance to human breast cancer cells. Int J Radiat Biol. 2011;87:263–73. 10.3109/09553002.2011.530335. 21067293

[21] Wu J, Chen Y, Lu L-Y, Wu Y, Paulsen MT, Ljungman M, et al. Chfr and RNF8 synergistically regulate ATM activation. Nat Struct Mol Biol. 2011;18:761–8. 10.1038/nsmb.2078. 21706008 PMC3130800

[22] Lavin MF. Ataxia-telangiectasia: from a rare disorder to a paradigm for cell signalling and cancer. Nat Rev Mol Cell Biol. 2008;9:759–69. 10.1038/nrm2514. 18813293

[23] Khan MGM, Wang Y. Advances in the Current Understanding of How Low-Dose Radiation Affects the Cell Cycle. Cells. 2022;11:356. 10.3390/cells11030356. 35159169 PMC8834401

[24] Xie S, Hong Z, Li Y, Wang J, Wang J, Li S, et al. RNF216 Alleviates Radiation-Induced Apoptosis and DNA Damage Through Regulating Ubiquitination-Mediated Degradation of p53 in Glioblastoma. Mol Neurobiol. 2022;59:4703–17. 10.1007/s12035-022-02868-6. 35594003

[25] Luo H, Yount C, Lang H, Yang A, Riemer EC, Lyons K, et al. Activation of p53 with Nutlin-3a radiosensitizes lung cancer cells via enhancing radiation-induced premature senescence. Lung Cancer Amst Neth. 2013;81:167–73. 10.1016/j.lungcan.2013.04.017. PMC3739976 23683497

[26] Wiese C, Pierce AJ, Gauny SS, Jasin M, Kronenberg A. Gene conversion is strongly induced in human cells by double-strand breaks and is modulated by the expression of BCL-x(L). Cancer Res. 2002;62:1279–83. 11888891

[27] Dong X, Li X, Gan Y, Ding J, Wei B, Zhou L, et al. TRAF4-mediated ubiquitination-dependent activation of JNK/Bcl-xL drives radioresistance. Cell Death Dis. 2023;14:102. 10.1038/s41419-023-05637-y. 36765039 PMC9918491

[28] Wang SJ, Dougan SK, Dougan M. Immune mechanisms of toxicity from checkpoint inhibitors. Trends Cancer. 2023;9:543–53. 10.1016/j.trecan.2023.04.002. 37117135 PMC10330206

[29] Pauken KE, Torchia JA, Chaudhri A, Sharpe AH, Freeman GJ. Emerging concepts in PD-1 checkpoint biology. Semin Immunol. 2021;52:101480. 10.1016/j.smim.2021.101480. 34006473 PMC8545711

[30] Jing W, Wang G, Cui Z, Xiong G, Jiang X, Li Y, et al. FGFR3 Destabilizes PD-L1 via NEDD4 to Control T-cell-Mediated Bladder Cancer Immune Surveillance. Cancer Res. 2022;82:114–29. 10.1158/0008-5472.CAN-21-2362. 34753771

[31] Persaud A, Alberts P, Mari S, Tong J, Murchie R, Maspero E, et al. Tyrosine phosphorylation of NEDD4 activates its ubiquitin ligase activity. Sci Signal. 2014;7:ra95. 10.1126/scisignal.2005290. 25292214

[32] Augustin RC, Bao R, Luke JJ. Targeting Cbl-b in cancer immunotherapy. J Immunother Cancer. 2023;11:e006007. 10.1136/jitc-2022-006007. 36750253 PMC9906388

[33] Hu Q, Ye Y, Chan L-C, Li Y, Liang K, Lin A, et al. Oncogenic lncRNA downregulates cancer cell antigen presentation and intrinsic tumor suppression. Nat Immunol. 2019;20:835–51. 10.1038/s41590-019-0400-7. 31160797 PMC6619502

[34] Vitari AC, Leong KG, Newton K, Yee C, O’Rourke K, Liu J, et al. COP1 is a tumour suppressor that causes degradation of ETS transcription factors. Nature. 2011;474:403–6. 10.1038/nature10005. 21572435

[35] Wang X, Tokheim C, Gu SS, Wang B, Tang Q, Li Y, et al. In vivo CRISPR screens identify the E3 ligase Cop1 as a modulator of macrophage infiltration and cancer immunotherapy target. Cell. 2021;184:5357–e537422. 10.1016/j.cell.2021.09.006. 34582788 PMC9136996

[36] Yu C, Xue B, Li J, Zhang Q. Tumor cell-derived exosome RNF126 affects the immune microenvironment and promotes nasopharyngeal carcinoma progression by regulating PTEN ubiquitination. Apoptosis Int J Program Cell Death. 2022;27:590–605. 10.1007/s10495-022-01738-9. 35717659

[37] Yu N, Xue M, Wang W, Xia D, Li Y, Zhou X, et al. RNF168 facilitates proliferation and invasion of esophageal carcinoma, possibly via stabilizing STAT1. J Cell Mol Med. 2019;23:1553–61. 10.1111/jcmm.14063. 30506884 PMC6349343

[38] Gatti M, Pinato S, Maiolica A, Rocchio F, Prato MG, Aebersold R, et al. RNF168 promotes noncanonical K27 ubiquitination to signal DNA damage. Cell Rep. 2015;10:226–38. 10.1016/j.celrep.2014.12.021. 25578731

[39] Yang Y-G, Lindahl T, Barnes DE. Trex1 exonuclease degrades ssDNA to prevent chronic checkpoint activation and autoimmune disease. Cell. 2007;131:873–86. 10.1016/j.cell.2007.10.017. 18045533

[40] Nader GP, de F, Agüera-Gonzalez S, Routet F, Gratia M, Maurin M, Cancila V, et al. Compromised nuclear envelope integrity drives TREX1-dependent DNA damage and tumor cell invasion. Cell. 2021;184:5230–e524622. 10.1016/j.cell.2021.08.035. 34551315

[41] Tani T, Mathsyaraja H, Campisi M, Li Z-H, Haratani K, Fahey CG, et al. TREX1 Inactivation Unleashes Cancer Cell STING-Interferon Signaling and Promotes Antitumor Immunity. Cancer Discov. 2024;14:752–65. 10.1158/2159-8290.CD-23-0700. 38227896 PMC11062818

[42] Barker FG, Simmons ML, Chang SM, Prados MD, Larson DA, Sneed PK, et al. EGFR overexpression and radiation response in glioblastoma multiforme. Int J Radiat Oncol Biol Phys. 2001;51:410–8. 10.1016/s0360-3016(01)01609-1. 11567815

[43] Xue X, Kang J-B, Yang X, Li N, Chang L, Ji J, et al. An efficient strategy for digging protein-protein interactions for rational drug design - A case study with HIF-1α/VHL. Eur J Med Chem. 2022;227:113871. 10.1016/j.ejmech.2021.113871. 34638033

[44] Chen Y, Zhang M, Liu Z, Zhang N, Wang Q. Ursodeoxycholic Acid Platinum(IV) Conjugates as Antiproliferative and Antimetastatic Agents: Remodel the Tumor Microenvironment through Suppressing JAK2/STAT3 Signaling. J Med Chem. 2024;67:17551–67. 10.1021/acs.jmedchem.4c01549. 39292635

[45] Kong L, Zhou M, Yuan W, Wang Y, Liu X, Wang J, et al. Radiation-Enhanced CD24 Membrane Trafficking via GPI Anchoring Mediates Antitumor Immune Evasion. Cancer Res. 2026;86:1480–95. 10.1158/0008-5472.CAN-25-2616. 41512182 PMC13012240

[46] Li J-Y, Zhao Y, Gong S, Wang M-M, Liu X, He Q-M, et al. TRIM21 inhibits irradiation-induced mitochondrial DNA release and impairs antitumour immunity in nasopharyngeal carcinoma tumour models. Nat Commun. 2023;14:865. 10.1038/s41467-023-36523-y. 36797289 PMC9935546

[47] Li M, Gao F, Li X, Gan Y, Han S, Yu X, et al. Stabilization of MCL-1 by E3 ligase TRAF4 confers radioresistance. Cell Death Dis. 2022;13:1053. 10.1038/s41419-022-05500-6. 36535926 PMC9763423

[48] Liao J, Qing X, Li X, Gan Y, Wang R, Han S, et al. TRAF4 regulates ubiquitination-modulated survivin turnover and confers radioresistance. Int J Biol Sci. 2024;20:182–99. 10.7150/ijbs.87180. 38164179 PMC10750280

[49] Wang R, Yang X, Wong YH, Guo L, Wu C, Chan BP. PINCH-1-dependent regulation on tumor matrix microenvironment in pancreatic cancer. Biomaterials. 2026;330:124074. 10.1016/j.biomaterials.2026.124074. 41691796

[50] Shen S, Liu X, Guo Q, Liang Q, Wu J, Guan G, et al. Tumor microenvironment remodeling plus immunotherapy could be used in mesenchymal-like tumor with high tumor residual and drug resistant rate. Commun Biol. 2023;6:1281. 10.1038/s42003-023-05667-4. 38110614 PMC10728080

[51] Pandya P, Kublo L, Stewart-Ornstein J. p53 Promotes Cytokine Expression in Melanoma to Regulate Drug Resistance and Migration. Cells. 2022;11:405. 10.3390/cells11030405. 35159215 PMC8833998

[52] Nian Z, Dou Y, Wei H. p53: An Immune Ecosystem Manipulator. Clin Cancer Res. 2025;31:5120–7. 10.1158/1078-0432.CCR-24-3628. 41086053

[53] Cerami E, Gao J, Dogrusoz U, Gross BE, Sumer SO, Aksoy BA, et al. The cBio cancer genomics portal: an open platform for exploring multidimensional cancer genomics data. Cancer Discov. 2012;2:401–4. 10.1158/2159-8290.CD-12-0095. 22588877 PMC3956037

[54] Hu Q, Li L, Li Y, Zhang H, Deng T, Liu Y, et al. A structure-based virtual screening identifies a novel MDM2 antagonist in the activation of the p53 signaling and inhibition of tumor growth. Acta Pharmacol Sin. 2025;46:740–50. 10.1038/s41401-024-01394-6. 39384887 PMC11845602

[55] Prabakaran PJ, Javaid AM, Swick AD, Werner LR, Nickel KP, Sampene E, et al. Radiosensitization of Adenoid Cystic Carcinoma with MDM2 Inhibition. Clin Cancer Res Off J Am Assoc Cancer Res. 2017;23:6044–53. 10.1158/1078-0432.CCR-17-0969. PMC5641244 28659312

[56] Zhou J, Kryczek I, Li S, Li X, Aguilar A, Wei S, et al. The ubiquitin ligase MDM2 sustains STAT5 stability to control T cell-mediated antitumor immunity. Nat Immunol. 2021;22:460–70. 10.1038/s41590-021-00888-3. 33767425 PMC8026726

[57] Sha B, Sun Y, Zhao S, Li M, Huang W, Li Z, et al. USP8 inhibitor-induced DNA damage activates cell cycle arrest, apoptosis, and autophagy in esophageal squamous cell carcinoma. Cell Biol Toxicol. 2023;39:2011–32. 10.1007/s10565-021-09686-x. 35022897

[58] Shi L, Lu J, Xia X, Liu X, Li H, Li X, et al. Clinically used drug arsenic trioxide targets XIAP and overcomes apoptosis resistance in an organoid-based preclinical cancer model. Chem Sci. 2024;15:8311–22. 10.1039/d4sc01294a. 38846391 PMC11151819

[59] Mehdizadeh K, Ataei F, Hosseinkhani S. Monitoring dimer structure orientation of full-length XIAP in living cells using a bioluminescence-based complementation assay. Int J Biol Macromol. 2025;312:143937. 10.1016/j.ijbiomac.2025.143937. 40334897

[60] Daoud M, Broxtermann PN, Schorn F, Werthenbach JP, Seeger JM, Schiffmann LM, et al. XIAP promotes melanoma growth by inducing tumour neutrophil infiltration. EMBO Rep. 2022;23:e53608. 10.15252/embr.202153608. 35437868 PMC9171690

[61] Hughes SA, Lin M, Weir A, Huang B, Xiong L, Chua NK, et al. Caspase-8-driven apoptotic and pyroptotic crosstalk causes cell death and IL-1β release in X-linked inhibitor of apoptosis (XIAP) deficiency. EMBO J. 2023;42:e110468. 10.15252/embj.2021110468. 36647737 PMC9975961

[62] Xi Y, Chen L, Tang J, Yu B, Shen W, Niu X. Amplifying eat me signal by immunogenic cell death for potentiating cancer immunotherapy. Immunol Rev. 2024;321:94–114. 10.1111/imr.13251. 37550950

[63] Li H, Wang M, Huang B, Zhu S-W, Zhou J-J, Chen D-R, et al. Theranostic near-infrared-IIb emitting nanoprobes for promoting immunogenic radiotherapy and abscopal effects against cancer metastasis. Nat Commun. 2021;12:7149. 10.1038/s41467-021-27485-0. 34887404 PMC8660774

[64] Song H, Sun H, He N, Xu C, Wang Y, Du L, et al. Gadolinium-based ultra-small nanoparticles augment radiotherapy-induced T-cell response to synergize with checkpoint blockade immunotherapy. Nanoscale. 2022;14:11429–42. 10.1039/d2nr02620a. 35904053

[65] Lyu M, Zhang T, Bao Z, Li P, Chen M, Quan H, et al. In situ forming AIEgen-alginate hydrogel for remodeling tumor microenvironment to boost FLASH immunoradiotherapy. Biomaterials. 2025;320:123281. 10.1016/j.biomaterials.2025.123281. 40138965

[66] Chen Z, Wang W, Hou J, Gao C, Song M, Zhao Z, et al. NEDD4L contributes to ferroptosis and cell growth inhibition in esophageal squamous cell carcinoma by facilitating xCT ubiquitination. Cell Death Discov. 2024;10:473. 10.1038/s41420-024-02243-5. 39557844 PMC11574128

[67] Shin S, Bae G-H, Park JD, Koh E-Y, Ko S, Han J, et al. Transforming lipid nanoparticles into radio-activatable therapeutics through synergistic ferroptosis for enhanced cancer radiotherapy. Biomaterials. 2026;330:124002. 10.1016/j.biomaterials.2026.124002. 41570670

[68] Lei G, Mao C, Yan Y, Zhuang L, Gan B. Ferroptosis, radiotherapy, and combination therapeutic strategies. Protein Cell. 2021;12:836–57. 10.1007/s13238-021-00841-y. 33891303 PMC8563889

[69] Wei G-X, Zhao Y-Q, Tang R, Shi H-S, Li Z-P. Ferroptosis at the intersection of radiotherapy and immunotherapy. Cancer Metastasis Rev. 2025;44:89. 10.1007/s10555-025-10306-x. 41369990

[70] Nguyen T, Nioi P, Pickett CB. The Nrf2-antioxidant response element signaling pathway and its activation by oxidative stress. J Biol Chem. 2009;284:13291–5. 10.1074/jbc.R900010200. 19182219 PMC2679427

[71] Da C, Pu J, Liu Z, Wei J, Qu Y, Wu Y, et al. HACE1-mediated NRF2 activation causes enhanced malignant phenotypes and decreased radiosensitivity of glioma cells. Signal Transduct Target Ther. 2021;6:399. 10.1038/s41392-021-00793-z. 34815381 PMC8611003

[72] McDonald JT, Kim K, Norris AJ, Vlashi E, Phillips TM, Lagadec C, et al. Ionizing radiation activates the Nrf2 antioxidant response. Cancer Res. 2010;70:8886–95. 10.1158/0008-5472.CAN-10-0171. 20940400 PMC2970706

[73] Castillo-Lluva S, Tan C-T, Daugaard M, Sorensen PHB, Malliri A. The tumour suppressor HACE1 controls cell migration by regulating Rac1 degradation. Oncogene. 2013;32:1735–42. 10.1038/onc.2012.189. 22614015

[74] Schaer DJ, Schulthess-Lutz N, Baselgia L, Kunasingam K, Humar R, Hansen K, et al. Stress-NRF2 response axis polarizes tumor macrophages and undermines immunotherapy. J Immunother Cancer. 2025;13:e013063. 10.1136/jitc-2025-013063. 41176316 PMC12581087

[75] Patel R, Saab K, Luo L, Ma Y, Osman RA, Williams NT, et al. Nrf2 Hyperactivation as a Driver of Radiotherapy Resistance and Suppressed Antitumor Immunity in Head and Neck Squamous Cell Carcinoma. Clin Cancer Res Off J Am Assoc Cancer Res. 2025;31:4184–95. 10.1158/1078-0432.CCR-25-0112. PMC12428276 40742317

[76] Jo Y, Shim JA, Jeong JW, Kim H, Lee SM, Jeong J, et al. Targeting ROS-sensing Nrf2 potentiates anti-tumor immunity of intratumoral CD8 + T and CAR-T cells. Mol Ther J Am Soc Gene Ther. 2024;32:3879–94. 10.1016/j.ymthe.2024.08.019. PMC11573615 39169624

[77] Che Y, Jiang D, Xu L, Sun Y, Wu Y, Liu Y, et al. The Clinical Prediction Value of the Ubiquitination Model Reflecting the Immune Traits in LUAD. Front Immunol. 2022;13:846402. 10.3389/fimmu.2022.846402. 35281055 PMC8913715

[78] Lin X, Zhao S, Li L, Huang Y, Zhong Q, Luo H, et al. Prognostic model of ubiquitination-related genes in ovarian cancer based on transcriptomic analysis and experimental validation. Front Immunol. 2025;16:1654180. 10.3389/fimmu.2025.1654180. 40990020 PMC12450667

[79] Sun D, Duan X, Li N, Qiao O, Hou Y, Ma Z, et al. Construction of ubiquitination-related risk model for predicting prognosis in lung adenocarcinoma. Sci Rep. 2025;15:11787. 10.1038/s41598-025-92177-4. 40189665 PMC11973225

[80] Guo Y, Wu Z, Cen K, Bai Y, Dai Y, Mai Y, et al. Establishment and validation of a ubiquitination-related gene signature associated with prognosis in pancreatic duct adenocarcinoma. Front Immunol. 2023;14:1171811. 10.3389/fimmu.2023.1171811. 37359528 PMC10289160

[81] Shi J, Zhang Z, Chen H-Y, Yao Y, Ke S, Yu K, et al. Targeting the TRIM21-PD-1 axis potentiates immune checkpoint blockade and CAR-T cell therapy. Mol Ther J Am Soc Gene Ther. 2025;33:1073–90. 10.1016/j.ymthe.2025.01.047. PMC11897759 39905727

[82] Wang X, Zhao Y, Yang X, Liu T, Zhou W, Niu S, et al. Radiation-induced nuclear translocation of NPRL2 hijacks E3 ubiquitin ligases to enhance DNA repair via the AMPK/WDR24 axis, contributing to CRC radioresistance. Acta Pharm Sin B. 2026;16:252–69. 10.1016/j.apsb.2025.10.039. 41584340 PMC12828144

[83] Gstalder C, Liu D, Miao D, Lutterbach B, DeVine AL, Lin C, et al. Inactivation of Fbxw7 Impairs dsRNA Sensing and Confers Resistance to PD-1 Blockade. Cancer Discov. 2020;10:1296–311. 10.1158/2159-8290.CD-19-1416. 32371478 PMC8802534

[84] Vanpouille-Box C, Alard A, Aryankalayil MJ, Sarfraz Y, Diamond JM, Schneider RJ, et al. DNA exonuclease Trex1 regulates radiotherapy-induced tumour immunogenicity. Nat Commun. 2017;8:15618. 10.1038/ncomms15618. 28598415 PMC5472757

[85] Spaas M, Sundahl N, Kruse V, Rottey S, De Maeseneer D, Duprez F, et al. Checkpoint Inhibitors in Combination With Stereotactic Body Radiotherapy in Patients With Advanced Solid Tumors: The CHEERS Phase 2 Randomized Clinical Trial. JAMA Oncol. 2023;9:1205. 10.1001/jamaoncol.2023.2132. 37410476 PMC10326732

[86] Xiu Z, Sun T, Yang Y, He Y, Yang S, Xue X et al. B <>Wang editor 2022 Curcumin Enhanced Ionizing Radiation-Induced Immunogenic Cell Death in Glioma Cells through Endoplasmic Reticulum Stress Signaling Pathways. Oxid Med Cell Longev 2022 5424411 10.1155/2022/5424411. PMC9553401 36238646

[87] Zhang J, Bu X, Wang H, Zhu Y, Geng Y, Nihira NT, et al. Author Correction: Cyclin D-CDK4 kinase destabilizes PD-L1 via cullin 3-SPOP to control cancer immune surveillance. Nature. 2019;571:E10. 10.1038/s41586-019-1351-8. 31270456

[88] Yu Z, Wu X, Zhu J, Yan H, Li Y, Zhang H, et al. BCLAF1 binds SPOP to stabilize PD-L1 and promotes the development and immune escape of hepatocellular carcinoma. Cell Mol Life Sci. 2024;81:82. 10.1007/s00018-024-05144-z. 38340178 PMC10858942

[89] Gao K, Shi Q, Gu Y, Yang W, He Y, Lv Z, et al. SPOP mutations promote tumor immune escape in endometrial cancer via the IRF1–PD-L1 axis. Cell Death Differ. 2023;30:475–87. 10.1038/s41418-022-01097-7. 36481790 PMC9950446

[90] Zhao Q, Li C, Zhang M, Gao T, Wang Z, Li Z, et al. Tumor PD-L1 induces β2m ubiquitylation and degradation for cancer cell immune evasion. Cell Res. 2026;36:38–50. 10.1038/s41422-025-01205-5. 41478857 PMC12765868

[91] Chen X, Lu Q, Zhou H, Liu J, Nadorp B, Lasry A, et al. A membrane-associated MHC-I inhibitory axis for cancer immune evasion. Cell. 2023;186:3903–e392021. 10.1016/j.cell.2023.07.016. 37557169 PMC10961051

[92] Fleischmann J, Hildebrand LS, Kuhlmann L, Fietkau R, Distel LV. The Effect of Xevinapant Combined with Ionizing Radiation on HNSCC and Normal Tissue Cells and the Impact of Xevinapant on Its Targeted Proteins cIAP1 and XIAP. Cells. 2023;12:1653. 10.3390/cells12121653. 37371123 PMC10297233

[93] Wu Q, Chen J, Berglund AE, Du D, Macaulay RJ, Etame AB. The reprogramming impact of SMAC-mimetic on glioblastoma stem cells and the immune tumor microenvironment evolution. J Exp Clin Cancer Res. 2025;44:191. 10.1186/s13046-025-03452-1. 40616096 PMC12231904

[94] Tao Y, Sun X-S, Pointreau Y, Le Tourneau C, Sire C, Kaminsky M-C, et al. Extended follow-up of a phase 2 trial of xevinapant plus chemoradiotherapy in high-risk locally advanced squamous cell carcinoma of the head and neck: a randomised clinical trial. Eur J Cancer. 2023;183:24–37. 10.1016/j.ejca.2022.12.015. 36796234

[95] Vugmeyster Y, Ravula A, Rouits E, Diderichsen PM, Kleijn HJ, Koenig A, et al. Model-Informed Selection of the Recommended Phase III Dose of the Inhibitor of Apoptosis Protein Inhibitor, Xevinapant, in Combination with Cisplatin and Concurrent Radiotherapy in Patients with Locally Advanced Squamous Cell Carcinoma of the Head and Neck. Clin Pharmacol Ther. 2024;115:52–61. 10.1002/cpt.3065. 37777832

[96] Bourhis J, Licitra LF, Burtness B, Psyrri A, Haddad R, Harrington K, et al. Xevinapant or Placebo Plus Platinum-Based Chemoradiotherapy in Unresected Locally Advanced Squamous Cell Carcinoma of the Head and Neck (TrilynX): A Randomized, Phase III Study. J Clin Oncol. 2025;43:3209–20. 10.1200/JCO-25-00272. 40902136 PMC12509437

[97] Schmitt NC, Stokes WA, Bates JE, Kinney BLC, Remick J, McDonald MW, et al. Early-Phase Trial of IAP Antagonist Tolinapant and Definitive Radiation in Cisplatin-Ineligible Patients with Advanced Head and Neck Cancer. Clin Cancer Res Off J Am Assoc Cancer Res. 2025;31:2910–8. 10.1158/1078-0432.CCR-25-0429. PMC12263315 40353748

[98] Afsahi A, Silvestri CM, Moore AE, Graham CF, Bacchiochi K, St-Jean M, et al. LCL161 enhances expansion and survival of engineered anti-tumor T cells but is restricted by death signaling. Front Immunol. 2023;14:1179827. 10.3389/fimmu.2023.1179827. 37138866 PMC10150108

[99] Johnson ML, Patel MR, Aljumaily R, Jones SF, Burris Iii HA, Spigel DR. A Phase Ib Dose-Escalation Study of LCL161 Plus Oral Topotecan for Patients With Relapsed/Refractory Small Cell Lung Cancer and Select Gynecologic Malignancies. Oncologist. 2023;28:640–e559. 10.1093/oncolo/oyad029. 37129455 PMC10322128

[100] Zhang X, Wen X, Peng R, Pan Q, Weng D, Ma Y, et al. A first-in-human phase I study of a novel MDM2/p53 inhibitor alrizomadlin in advanced solid tumors. ESMO Open. 2024;9:103636. 10.1016/j.esmoop.2024.103636. 39002360 PMC11452328

[101] Reijers ILM, Menzies AM, Versluis JM, Saw RPM, van Houdt WJ, Kapiteijn E et al. The impact of response-directed surgery and adjuvant therapy on long-term survival after neoadjuvant ipilimumab plus nivolumab in stage III melanoma: Three-year data of PRADO and OpACIN-neo. 10.1016/j.ejca.2024.115141 39602990

[102] Fang DD, Tang Q, Kong Y, Wang Q, Gu J, Fang X, et al. MDM2 inhibitor APG-115 synergizes with PD-1 blockade through enhancing antitumor immunity in the tumor microenvironment. J Immunother Cancer. 2019;7:327. 10.1186/s40425-019-0750-6. 31779710 PMC6883539

[103] Gounder MM, Bauer TM, Schwartz GK, Weise AM, LoRusso P, Kumar P, et al. A First-in-Human Phase I Study of Milademetan, an MDM2 Inhibitor, in Patients With Advanced Liposarcoma, Solid Tumors, or Lymphomas. J Clin Oncol Off J Am Soc Clin Oncol. 2023;41:1714–24. 10.1200/JCO.22.01285. PMC10022862 36669146

[104] Stein EM, DeAngelo DJ, Chromik J, Chatterjee M, Bauer S, Lin C-C, et al. Results from a First-in-Human Phase I Study of Siremadlin (HDM201) in Patients with Advanced Wild-Type TP53 Solid Tumors and Acute Leukemia. Clin Cancer Res Off J Am Assoc Cancer Res. 2022;28:870–81. 10.1158/1078-0432.CCR-21-1295. PMC9377734 34862243

[105] Ho JNHG, Schmidt D, Lowinus T, Ryoo J, Dopfer E-P, Gonzalo Núñez N, et al. Targeting MDM2 enhances antileukemia immunity after allogeneic transplantation via MHC-II and TRAIL-R1/2 upregulation. Blood. 2022;140:1167–81. 10.1182/blood.2022016082. 35853161 PMC9461473

[106] Phelps D, Bondra K, Seum S, Chronowski C, Leasure J, Kurmasheva RT, et al. Inhibition of MDM2 by RG7388 confers hypersensitivity to X-radiation in xenograft models of childhood sarcoma. Pediatr Blood Cancer. 2015;62:1345–52. 10.1002/pbc.25465. 25832557 PMC4563820

[107] Pervushin NV, Nilov DK, Pushkarev SV, Shipunova VO, Badlaeva AS, Yapryntseva MA, et al. BH3-mimetics or DNA-damaging agents in combination with RG7388 overcome p53 mutation-induced resistance to MDM2 inhibition. Apoptosis Int J Program Cell Death. 2024;29:2197–213. 10.1007/s10495-024-02014-8. PMC11550243 39222276

[108] Perez B, Aljumaily R, Marron TU, Shafique MR, Burris H, Iams WT, et al. Phase I study of peposertib and avelumab with or without palliative radiotherapy in patients with advanced solid tumors. ESMO Open. 2024;9:102217. 10.1016/j.esmoop.2023.102217. 38320431 PMC10937199

[109] Carr MI, Chiu L-Y, Guo Y, Xu C, Lazorchak AS, Yu H, et al. DNA-PK Inhibitor Peposertib Amplifies Radiation-Induced Inflammatory Micronucleation and Enhances TGFβ/PD-L1 Targeted Cancer Immunotherapy. Mol Cancer Res MCR. 2022;20:568–82. 10.1158/1541-7786.MCR-21-0612. 34980594 PMC9381110

[110] Haines E, Nishida Y, Carr MI, Montoya RH, Ostermann LB, Zhang W, et al. DNA-PK inhibitor peposertib enhances p53-dependent cytotoxicity of DNA double-strand break inducing therapy in acute leukemia. Sci Rep. 2021;11:12148. 10.1038/s41598-021-90500-3. 34108527 PMC8190296

[111] Schmidt ML, Donninger H, Clark GJ. Ras regulates SCF(β-TrCP) protein activity and specificity via its effector protein NORE1A. J Biol Chem. 2014;289:31102–10. 10.1074/jbc.M114.594283. 25217643 PMC4223314

[112] Weathington NM, Mallampalli RK. Emerging therapies targeting the ubiquitin proteasome system in cancer. J Clin Invest. 2014;124:6–12. 10.1172/JCI71602. 24382383 PMC3871250

[113] Schneekloth JS, Fonseca FN, Koldobskiy M, Mandal A, Deshaies R, Sakamoto K, et al. Chemical genetic control of protein levels: selective in vivo targeted degradation. J Am Chem Soc. 2004;126:3748–54. 10.1021/ja039025z. 15038727

[114] Crowe C, Nakasone MA, Chandler S, Craigon C, Sathe G, Tatham MH, et al. Mechanism of degrader-targeted protein ubiquitinability. Sci Adv. 2024;10:eado6492. 10.1126/sciadv.ado6492. 39392888 PMC11468923

[115] Ma L, Wang J, Zhang Y, Fang F, Ling J, Chu X, et al. BRD4 PROTAC degrader MZ1 exerts anticancer effects in acute myeloid leukemia by targeting c-Myc and ANP32B genes. Cancer Biol Ther. 2022;23:1–15. 10.1080/15384047.2022.2125748. 36170346 PMC9543111

[116] Gough SM, Flanagan JJ, Teh J, Andreoli M, Rousseau E, Pannone M, et al. Oral Estrogen Receptor PROTAC Vepdegestrant (ARV-471) Is Highly Efficacious as Monotherapy and in Combination with CDK4/6 or PI3K/mTOR Pathway Inhibitors in Preclinical ER+ Breast Cancer Models. Clin Cancer Res Off J Am Assoc Cancer Res. 2024;30:3549–63. 10.1158/1078-0432.CCR-23-3465. PMC11325148 38819400

[117] Campone M, De Laurentiis M, Jhaveri K, Hu X, Ladoire S, Patsouris A, et al. Vepdegestrant, a PROTAC Estrogen Receptor Degrader, in Advanced Breast Cancer. N Engl J Med. 2025;393:556–68. 10.1056/NEJMoa2505725. 40454645

[118] Snyder LB, Neklesa TK, Willard RR, Gordon DA, Pizzano J, Vitale N, et al. Preclinical Evaluation of Bavdegalutamide (ARV-110), a Novel PROteolysis TArgeting Chimera Androgen Receptor Degrader. Mol Cancer Ther. 2025;24:511–22. 10.1158/1535-7163.MCT-23-0655. 39670468 PMC11962395

[119] Khan S, Zhang X, Lv D, Zhang Q, He Y, Zhang P, et al. A selective BCL-XL PROTAC degrader achieves safe and potent antitumor activity. Nat Med. 2019;25:1938–47. 10.1038/s41591-019-0668-z. 31792461 PMC6898785

[120] Feldman T, Marchi E, Smith SD, Olszewski AJ, Huen AO, Epstein-Peterson ZD, et al. Safety, Pharmacokinetics, Pharmacodynamics and Clinical Activity of KT-333, a Targeted Protein Degrader of STAT3, in Patients with Relapsed or Refractory Lymphomas, Leukemia, and Solid Tumors. Blood. 2024;144:4433–4433. 10.1182/blood-2024-210301.

[121] Oleinikovas V, Gainza P, Ryckmans T, Fasching B, Thomä NH. From Thalidomide to Rational Molecular Glue Design for Targeted Protein Degradation. Annu Rev Pharmacol Toxicol. 2024;64:291–312. 10.1146/annurev-pharmtox-022123-104147. 37585660

[122] Fischer ES, Böhm K, Lydeard JR, Yang H, Stadler MB, Cavadini S, et al. Structure of the DDB1-CRBN E3 ubiquitin ligase in complex with thalidomide. Nature. 2014;512:49–53. 10.1038/nature13527. 25043012 PMC4423819

[123] Richardson PG, Trudel S, Popat R, Mateos M-V, Vangsted AJ, Ramasamy K, et al. Mezigdomide plus Dexamethasone in Relapsed and Refractory Multiple Myeloma. N Engl J Med. 2023;389:1009–22. 10.1056/NEJMoa2303194. 37646702

[124] Lu SX, De Neef E, Thomas JD, Sabio E, Rousseau B, Gigoux M, et al. Pharmacologic modulation of RNA splicing enhances anti-tumor immunity. Cell. 2021;184:4032–e404731. 10.1016/j.cell.2021.05.038. 34171309 PMC8684350

[125] Xu Y, Spear S, Ma Y, Lorentzen MP, Gruet M, McKinney F, et al. Pharmacological depletion of RNA splicing factor RBM39 by indisulam synergizes with PARP inhibitors in high-grade serous ovarian carcinoma. Cell Rep. 2023;42:113307. 10.1016/j.celrep.2023.113307. 37858464

